# Understanding the relationship between stay-at-home measures and vaccine shortages: a conventional, heterogeneous, and fractional dynamic approach

**DOI:** 10.1186/s41043-024-00505-7

**Published:** 2024-02-29

**Authors:** Mohammad Sharif Ullah, Md. Kamrujjaman, K. M. Ariful Kabir

**Affiliations:** 1 Department of Mathematics, Feni University, Feni, Bangladesh; 2 Dhaka University, Dhaka, 1000 Bangladesh; 3 Department of Mathematics, Bangladesh University of Engineering and Technology, Dhaka, Bangladesh

**Keywords:** Non-pharmaceutical interventions, Vaccine shortage, Multi-waving, Network analysis, Memory effect

## Abstract

In light of the global prevalence of a highly contagious respiratory disease, this study presents a novel approach to address the pressing and unanticipated issues by introducing a modified vaccination and lockdown-centered epidemic model. The rapid spread of the disease is attributed to viral transmissibility, the emergence of new strains (variants), lack of immunization, and human unawareness. This study aims to provide policymakers with crucial insights for making informed decisions regarding lockdown strategies, vaccine availability, and other control measures. The research adopts three types of models: deterministic, heterogeneous, and fractional-order dynamics, on both theoretical and numerical approaches. The heterogeneous network considers varying connectivity and interaction patterns among individuals, while the ABC fractional-order derivatives analyze the impact of integer-order control in different semi-groups. An extensive theoretical analysis is conducted to validate the proposed model. A comprehensive numerical investigation encompasses deterministic, stochastic, and ABC fractional-order derivatives, considering the combined effects of an effective vaccination program and non-pharmaceutical interventions, such as lockdowns and shutdowns. The findings of this research are expected to be valuable for policymakers in different countries, helping them implement dynamic strategies to control and eradicate the epidemic effectively.

## Introduction

In the sense of controlling lethal disease, due to rapid spreading characteristics ‍and lack of proper treatment, the lockdown/shutdown policy became a common worldwide strategy to control its viral transmissibility. On top of that, vaccine shortages are fundamental reasons to impose the lockdown policy. Instead of economic loss, the lockdowns with varying levels assist policymakers in minimizing people’s lives if the lockdown maintenance factor works as expected. In addition, if there exists a considerable vaccine shortage worldwide, except for a few rich countries, these countries’ policymakers are frightened to minimize vaccine shortage for their people. Also, the vaccine efficacy rate significantly impacts talking about vaccination. As a result, the disease spreads worldwide within a very short period and harms humankind. To attain more realistic epidemic dynamics where vaccine shortage and lockdown policy aspects might happen, we considered a modified epidemic SEIR (susceptible-exposed-infected recovered) model [1–3].

The origins of mathematical modeling of infectious diseases may be traced back to the early 1900s [4]. With a mounting threat of population diseases in humanity, disease modeling exhibition has become a significant part of epidemic control [1, 5–7]. The mathematical model will help us compute potential facilitation impacts, how infectious diseases evolve, and forecast an epidemic or pandemic. In this respect, numerous lockdown, quarantine, awareness, and vaccination models have developed with time to elucidate disease dynamics. To reflect the behavioral dynamics of economic shutdowns and shield immunity during the pandemic time, Kabir and Tanimoto [8] propose an evolutionary game theory modeling technique. During an epidemic, Kabir et al. [9] briefly highlighted people’s employment of masks and similar protective practices that benefit the wearer and others. Alam et al. [10], Higazy et al. [11], Ullah et al. [12], and Higazy and Alyami [13] provided a comparative study of quarantine and isolation policy, transmission model in the context of the ABO blood group, all possible scenarios of a pandemic except the vaccination proportion, genetic algorithm-based control strategy model of epidemic transmission. However, the mentioned works did not consider the combined impact of lockdown and vaccine shortage, although they could present some exciting results. Considering the lack mentioned above works, we offer the combined effects of lockdown and vaccine shortage using a mathematical epidemiological model to guide this research work.

One of the most universally used methods for controlling the disease is the vaccination program to control and eradicate the contagious disease. It can provide an effective means to prevent communicable diseases and is critical for executing public health policies. On top of that, it deliberates direct protection to individuals, reduces the spread of infection, and provides herd protection to the population [14]. Most vaccination programs are optional, and individuals may choose whether or not to be vaccinated [15, 16]. However, many vaccine programs [17–19] have been found about mass vaccination strategies and forced vaccine programs. Besides, traditional and long-term immunization techniques are also crucial; see [17, 18]. Moreover, vaccine shortage is among the most critical issues for controlling infectious diseases and impacts people’s vaccination decisions. A shortage is unlikely to occur if a sufficient supply of vaccinations shows a unique equilibrium.

Furthermore, if the supply of vaccines is unavailable against the demand, a deficiency could occur in balance despite scarcity being self-fulfilling. When this circumstance develops, many put off being vaccinated. Furthermore, it heightens public fear and anxiety amid an epidemic, influencing people’s vaccination decisions. In such a situation, the interest in the minds of those who have decided to take the vaccine will increase as many people have decided to take it. Chen [19] theoretically analyzes voluntary vaccinations and vaccine shortages. Li et al. [20], Kahwati et al. [21], Fairbrother et al. [22], and Allison et al. [23] briefly discussed the impact of vaccine shortage on different epidemic diseases.

Typically, conventional compartmental models [2, 8–10] examine infectious disease transmission dynamics in compact, homogeneous populations. The primary issue lies in the equitable exposure of all individuals to an infected individual. Consequently, a growing body of academic research has focused on using complex networks in epidemic modeling for several decades. There is a consensus among scholars that considering contact heterogeneity's impact becomes relevant when dealing with sufficiently large populations. Consequently, incorporating a complex network is employed in the epidemic models to represent the impact of contact dynamics accurately. In a complex network, each individual is represented as a network node, while the association between two individuals is represented as a connection between two nodes. There is a growing emphasis on the need for increased scholarly investigation into the dynamics of the propagation model within both homogeneous and heterogeneous networks, specifically about incorporating network topology [24–27]. Pastor-Satorras and Vespignani [28, 29] proposed a widely recognized epidemic model for complex networks, in which they presented empirical evidence for the absence of an epidemic threshold due to a diverse contact pattern. This study marked the first instance of such a finding. They achieved this by employing entirely separate epidemic propagation scenarios. However, as mentioned earlier, the research studies are dealt with in the SI [30] or SIR [27–29, 31, 32] network epidemic model, and there is currently a lack of research specifically focused on the impact of lockdown measures. In this study, we will examine and analyze the SEIR-based vaccination model, which is widely recognized, along with a lockdown component. This analysis will be conducted for the first time on two distinct complex networks: the Erdős–Rényi random network (referred to as ER-random) [33] and the Barabasi–Albert scale-free (SF) network (referred to as BA) [34].

Many mathematicians and researchers from various fields have recently focused on the theory of fractional calculus and fractional differential equations to describe the different real-world phenomena and dynamical behavior of the epidemic diseases model [11–13, 35–41]. Because the derivative is obtained from evaluating integral over the region, fractional-order precision overrides integer order due to its non-local nature and changes every moment. In contrast, the value of an integer-order derivative assessed at a point depends exclusively on that point. On top of that, it is said that fractional derivatives are advantageous for demonstrating numerous everyday issues because of memory and universal features [11–13, 35, 42–44]. Therefore, the significance and potential application rose daily as fractional-order differential equations added additional dimensions to the study of epidemiological models, and numerous research [45–49] have addressed the fractional-order differential model. This study uses the ABC fractional-order derivative method [48]. According to the above discussion, it is clear that there is still no extensive study that combined both lockdown and vaccination strategies on the framework of fractional-order derivative and various heterogeneous network perspectives. Thus, we are the first to amalgamate lockdown and vaccination strategies as a non- pharmaceutical and pharmaceutical provision on two frameworks: fractional-order derivative and heterogeneous network.

This study presents a comprehensive framework for elucidating the joint effects of lockdown measures and vaccination programs, with vaccine efficacy being influenced by their availability both conventionally and fractionally. Furthermore, we investigate a heterogeneous network to accurately depict how these combined measures influence individuals’ behavior during an epidemic in a society. The results of the fractional-order simulation reveal that a lower value of the fractional order leads to a delayed peak in the epidemic, and the average degree distribution illustrates the variation in infection and control strategies. The proposed model sheds light on how the final size of the epidemic is impacted by the reduced efficacy of vaccines and their shortage. Interestingly, it is observed that prolonged lockdown measures may lead individuals to feel more compelled to leave their homes, consequently accelerating the spread of the disease. In light of these findings, it becomes evident that the most dynamic and effective strategies for controlling the epidemic involve a combination of an efficient vaccination program and non-pharmaceutical interventions, such as lockdowns, shutdowns, and states of emergency. This holistic approach holds promise in curbing the spread of the disease and mitigating its impact on society.

### Model formulation

The current model is based on the standard SEIR model. The whole population is partitioned into nine subgroups: Susceptible $S\left( t \right)$, Lock-down $L\left( t \right)$, Exposed $E\left( t \right)$, Infected $I\left( t \right)$, Recovered $R\left( t \right)$, Vaccinated $V\left( t \right)$, Vaccinated exposed $E_{V} \left( t \right)$, Vaccinated infected $I_{V} \left( t \right)$ and Vaccinated Recovered $R_{V} \left( t \right).$

Therefore, the proposed vaccination model is represented by the subsequent system of nonlinear ordinary differential equations:

1 $$\begin{aligned} \frac{{{\text{d}}S}}{{{\text{d}}t}} & = - \beta S\left( t \right)\left( {I\left( t \right) + I_{V} \left( t \right)} \right) - \delta ~S\left( t \right) - lS\left( t \right) + l_{d} L\left( t \right), \\ \frac{{{\text{d}}L}}{{{\text{d}}t}} & = lS\left( t \right) - \left( {1 - q} \right)\beta L\left( t \right)\left( {I\left( t \right) + I_{V} \left( t \right)} \right) - l_{d} L\left( t \right), \\ \frac{{{\text{d}}E}}{{{\text{d}}t}} & = \beta S\left( t \right)\left( {I\left( t \right) + I_{V} \left( t \right)} \right) + \left( {1 - q} \right)\beta L\left( t \right)\left( {I\left( t \right) + I_{V} \left( t \right)} \right)~ - \alpha E\left( t \right), \\ \frac{{{\text{d}}I}}{{{\text{d}}t}} & = \alpha E\left( t \right) - \gamma I\left( t \right), \\ \frac{{{\text{d}}R}}{{{\text{d}}t}} & = \gamma I\left( t \right), \\ \frac{{{\text{d}}V}}{{{\text{d}}t}} & = \delta ~S\left( t \right) - \left( {1 - \eta } \right)\beta V\left( t \right)\left( {I\left( t \right) + I_{V} \left( t \right)} \right), \\ \frac{{{\text{d}}E_{V} }}{{{\text{d}}t}} & = \left( {1 - \eta } \right)\beta V\left( t \right)\left( {I\left( t \right) + I_{V} \left( t \right)} \right) - \alpha E_{V} \left( t \right), \\ \frac{{{\text{d}}I_{V} }}{{{\text{d}}t}} & = \alpha E_{V} \left( t \right) - \gamma I_{V} \left( t \right), \\ \frac{{{\text{d}}R_{V} }}{{{\text{d}}t}} & = \gamma I_{V} \left( t \right). \\ \end{aligned}$$

The total population,

2 $$N\left( t \right) = S\left( t \right) + L\left( t \right) + E\left( t \right) + I\left( t \right) + R\left( t \right) + V\left( t \right) + E_{V} \left( t \right) + I_{V} \left( t \right) + R_{V} \left( t \right).$$

Let us assume that if ${\varvec{x}}$ is the state variable vector, then we can write,

$${\varvec{x}} = \left( {S\left( t \right), L\left( t \right), E\left( t \right) ,I\left( t \right), R\left( t \right), V\left( t \right), E_{V} \left( t \right),I_{V} \left( t \right), R_{V} \left( t \right)} \right)^{\prime }$$ and $$f:{\varvec{R}}^{9} \to {\varvec{R}}^{9} .$$

The suggested model's right side (Eq. 1) is thus a continuously differentiable function on ${\varvec{R}}^{9}$. A unique elucidation of Eq. (1) occurs at every initial condition and continues for the maximum existence interval [49]. As a result, the suggested model has a clear biological significance. The model’s solution is also positive $\forall t \ge 0$ and bounded by the entire population $N\left( t \right)$ according to [2, 11] (Eq. 1–2). Therefore, each compartment is regarded as one of the nine potential states at any time.

### Vaccine shortage

If $V_{o}$ is the amount of available vaccine, then

2.1 $$\delta = \left\{ {\begin{array}{*{20}l} 0 \hfill & {i{\text{f}}\quad V\left( t \right) > V_{o} ,} \hfill \\ \delta \hfill & {{\text{otherwise}}{.}} \hfill \\ \end{array} } \right.$$

(i) *Susceptible individuals, *$S\left( t \right)$ In the beginning, the susceptible part of the overall population is exposed to the infected individuals (first equation of 1). Those who lose immunity due to an earlier infection add to the vulnerable group and are reduced through vaccination (moving to class V with an efficacy rate $\left( {1 - \eta } \right)$ at the rate,$\delta$), following the lockdown level $l,$ infection (moving to class E).
(ii) *Lockdown individuals,* $L\left( t \right)$ Lockdown state members are the individuals who have adhered to the lockdown-related guidelines. It refers to susceptible people who stay at home and are not infected by the virus. The lockdown maintenance or obedient factor $\left( {1 - q} \right)$ has a crucial role in the prevention of infection. Here, $q = 0$ indicates that no people maintain lockdown rules, and $q = 1$, the lockdown policy works appropriately. Using the Heaviside function, we assess the lockdown open and shut mechanism.
  2.2 $$l\left[ {d_{{{\text{start}}}} ,d_{{{\text{end}}}} } \right] = \left\{ {\begin{array}{*{20}c} {0,t \notin \left[ {d_{{{\text{start}}}} ,d_{{{\text{end}}}} } \right],} \\ {1,t \in \left[ {d_{{{\text{start}}}} ,d_{{{\text{end}}}} } \right].} \\ \end{array} } \right.$$

where, $d_{start} =$ lockdown starting time, and $d_{end} =$ lockdown ending time.

(iii) *Exposed individuals,*$E\left( t \right)$ Infected people who are still vulnerable after being vaccinated increase the number of the exposed and not vaccinated yet; also, the lockdown maintenance or obedience factor. The onset of infection reduces the exposed population (moving to class $I\left( t \right)$) at the rate $\alpha$. During the exposed phase, humans maintain a low infectivity level. This compartment technically reflects the slightly infectious stage.
(iv) *Infected individuals,* $I\left( t \right)$ A fraction of exposed people evolve an infected class at the rate $\alpha$ increases the population of an infective class. Recovery from the disease at the rate $\gamma$ reduces the number of infective persons.
(v) *Recovered individuals,*$R\left( t \right)$ The recovered population increased from infective class at the rate $\gamma$.
(vi) *Vaccinated individuals,* $V\left( t \right)$ The number of people who have been vaccinated has grown as more people have been immunized. The vaccinated people were reduced by a factor ($1 - \eta$)$\beta$ with $0 \le \eta \le 1$.
(vii) *Vaccinated exposed individuals,*$E_{v} \left( t \right)$ The vaccinated exposed population increases if the mass susceptible individuals take part under the vaccinated program and are reduced by the onset of infection (moving to class $I_{V} \left( t \right)$) at the rate $\alpha$.
(viii) *Vaccinated infected individuals,*$I_{V} \left( t \right)$: The population of vaccinated infective class increased by a fraction of vaccinated exposed individuals becoming infective class at the rate $\alpha$. The vaccinated infected individuals are reduced by recovery from the disease at the rate $\gamma$.
(ix) *Vaccinated recovered individuals,*$R_{V} \left( t \right)$ The recovered population increased from the vaccinated infective class at the rate $\gamma$.

Table 1 explains the biological significance of the parameters and the values preferred for them.

**Table 1:** List of parameters, variables, and their biological meanings

Notation	Meaning	Value	Reference
$\beta$	Transmission rate	1.0	[15]
$\delta$	Vaccination rate	0.0–1.0	(Varied)
$l$	Lockdown level	0.0–0.9	(Varied)
${l}_{d}$	Lockdown day	–	(Varied)
$q (0\le q\le 1)$	Lockdown maintenance factor	0.0–1.0	Estimated
$\alpha$	Infection rate	1/5	[15]
$\gamma$	Recovery rate	0.1	Estimated
$1-\eta (0\le \eta \le 1)$	Vaccine efficacy	0.0–1.0	(Varied)
$S\left(t\right)$	Number of susceptible individuals	0.9998	Estimated
$L(t)$	Lockdown state	0.0	Estimated
$E(t)$	Number of exposed individuals	0.0	Estimated
$I(t)$	Number of infected individuals	0.0001	Estimated
$R(t)$	Number of recovered individuals	0.0	Estimated
$V(t)$	Number of vaccinated individuals	0.0	Estimated
${E}_{V}(t)$	Number of vaccinated exposed individuals	0.0	Estimated
${I}_{V}(t)$	Number of vaccinated infected individuals	0.0001	Estimated
${R}_{V}(t)$	Number of vaccinated recovered individuals	0.0	Estimated

### Network-based epidemic model

We suggest a network-based $SLEIRVE_{V} I_{V} R_{V}$ epidemic model in this section for the first time. This section describes the $SEIR$ model with the vaccine compartment. The model presented in this research work is a mean-field numerical approach since the complex network is dynamic, and the linkages are constantly rewired throughout the dynamics of an epidemic. The network under analysis is designated $N$. It is believed that each site in $N$ is either unoccupied or employed by a single person and that each site in $N$ may choose just one state from the $S,L,E,I,R,V,E_{V} ,I_{V}$ and $R_{V}$. Additionally, $n$ groups are created for each state based on the degree of the site. $S_{k} ,L_{k} ,E_{k} ,I_{k} ,R_{k} ,V_{k} ,E_{k}^{V} ,I_{k}^{V}$ and $R_{k}^{V}$, for $k = 1,2,3, \ldots ,n$, the number $n$ represents the density of those with degree $k$ who are susceptible, in lockdown state, exposed, infected, vaccinated, vaccinated exposed, vaccinated infected, and vaccinated recovered. The pace at which each site’s status may change varies. Therefore, the mean-field equations are expressed as follows:

3 $$\begin{aligned} \frac{{{\text{d}}S_{k} \left( t \right)}}{{{\text{d}}t}} & = - \beta kS_{k} \left( t \right)\theta \left( t \right) - \delta S_{k} \left( t \right) - lS_{k} \left( t \right) + l_{d} L_{k} \left( t \right), \\ \frac{{{\text{d}}L_{k} \left( t \right)}}{{{\text{d}}t}} & = lS_{k} \left( t \right) - \left( {1 - q} \right)\beta kL_{k} \left( t \right)\theta \left( t \right) - l_{d} L_{k} \left( t \right), \\ \frac{{{\text{d}}E_{k} \left( t \right)}}{{{\text{d}}t}} & = \beta kS_{k} \left( t \right)\theta \left( t \right) + \left( {1 - q} \right)\beta kL_{k} \left( t \right)\theta \left( t \right) - \alpha E_{k} \left( t \right), \\ \frac{{{\text{d}}I_{k} \left( t \right)}}{{{\text{d}}t}} & = \alpha E_{k} \left( t \right) - \gamma I_{k} \left( t \right), \\ \frac{{{\text{d}}R_{k\left( t \right)} }}{{{\text{d}}t}} & = \gamma I_{k} \left( t \right), \\ \frac{{{\text{d}}V\left( t \right)}}{{{\text{d}}t}} & = \delta S_{k} \left( t \right) - \left( {1 - \eta } \right)\beta kV_{k} \left( t \right)\theta \left( t \right), \\ \frac{{{\text{d}}E_{k}^{V} \left( t \right)}}{{{\text{d}}t}} & = \left( {1 - \eta } \right)\beta kV_{k} \left( t \right)\theta \left( t \right) - \alpha E_{k}^{V} \left( t \right), \\ \frac{{{\text{d}}I_{k}^{V} \left( t \right)}}{{{\text{d}}t}} & = \alpha E_{k}^{V} \left( t \right) - \gamma I_{k}^{V} \left( t \right), \\ \frac{{{\text{d}}R_{k}^{V} \left( t \right)}}{{{\text{d}}t}} & = \gamma I_{k}^{V} \left( t \right). \\ \end{aligned}$$

If a link links to a site of degree $k$, the probability that it does so is proportional to degree distribution $\left( {k - 1} \right)P\left( k \right)$ [28, 29] for uncorrelated complex degree networks, then ${\Theta }\left( t \right)$ is represented as

4 $${\Theta }\left( t \right) = \frac{{\mathop \sum \nolimits_{k} \left( {k - 1} \right)P\left( k \right)\left( {I_{k} \left( t \right) + I_{k}^{V} \left( t \right)} \right)}}{k},$$

where $P\left( k \right) > 0,$ the degree distribution of the network, which satisfies the normalized equality $\mathop \sum \limits_{k = 1}^{n} \left( {k - 1} \right)P\left( k \right) = 1,\;{\text{and}}$ $\left\langle k \right\rangle = \mathop \sum \limits_{k = 1}^{n} \left( {k - 1} \right)P\left( k \right)$ symbolizes the average degree of the network.

The proposed lockdown-vaccination model (3) with Eq. (9) and the initial condition $S_{k} \left( 0 \right) = S_{k}^{0} ,L_{k} \left( 0 \right) = L_{k}^{0} ,E_{k} \left( 0 \right) = E_{k}^{0} ,I_{k} \left( 0 \right) = I_{k}^{0} ,R_{k} \left( 0 \right) = R_{k}^{0} ,V_{k} \left( 0 \right) = V_{k}^{0} ,V_{Ek} \left( 0 \right) = E_{Vk}^{0} ,I_{Vk} \left( 0 \right) = I_{Vk}^{0} ,R_{Vk} \left( 0 \right) = R_{Vk}^{0}$ must be satisfied inequality $0 < S_{k}^{0} + L_{k}^{0} + E_{k}^{0} + I_{k}^{0} + R_{k}^{0} + V_{k}^{0} + E_{{Vk}}^{0} + I_{{Vk}}^{0} + R_{{Vk}}^{0} \le 1,$ which describes the dynamics of the vaccinated model on uncorrelated networks with degree distribution $\left( {k - 1} \right)P\left( k \right)$. Any system (3) solution starting from the nonnegative cone $R_{ + }^{9n}$ is shown to stay nonnegative. Therefore, we examine the $R_{ + }^{9n}$ model (3) in the following sections. Additionally, combining the four equations in the model (3) results in

5 $$\frac{{\text{d}}}{{{\text{d}}t}}\left( {S_{k} + L_{k} + E_{k} + I_{k} + R_{k} + V_{k} + E_{k}^{V} + I_{k}^{V} + R_{k}^{V} } \right) = {\text{N}}_{{\text{k}}} ,$$

where $${\text{N}}_{{\text{k}}} = {\text{total population}}.$$

Therefore,

$$\mathop {\lim }\limits_{t \to \infty } {\text{sup}}\left[ {S_{k} \left( t \right) + L_{k} \left( t \right) + E_{k} \left( t \right) + I_{k} \left( t \right) + R_{k} \left( t \right) + V_{k} \left( t \right) + E_{k}^{V} \left( t \right) + I_{k}^{V} \left( t \right) + R_{k}^{V} \left( t \right)} \right] \le {\text{N}}_{{\text{k}}} .$$

As a result, the feasible compact region

6 $$\begin{aligned} {\Pi } & = \left\{ {S_{1} ,L_{1} ,E_{1} ,I_{1} ,R_{1} ,V_{1} ,E_{1}^{V} ,I_{1}^{V} ,R_{1}^{V} , \ldots ,S_{k} ,L_{k} ,E_{k} ,I_{k} ,R_{k} ,V_{k} ,E_{k}^{V} ,I_{k}^{V} ,R_{k}^{V} } \right\} \in R_{ + }^{9n} :S_{k} \left( t \right) + L_{k} \left( t \right) \\ & \quad + E_{k} \left( t \right) + I_{k} \left( t \right) + R_{k} \left( t \right) + V_{k} \left( t \right) + E_{k}^{V} \left( t \right) + I_{k}^{V} \left( t \right) + R_{k}^{k} \left( t \right) \le N_{k} , \\ \end{aligned}$$

contains a positive invariant concerning the model (3) that is included in the nonnegative cone of $R_{ + }^{9n}$ with $1 \le k \le n$.

### Epidemic model based on ABC fractional derivative

In recent decades, mathematical models of integer derivatives have progressed considerably due to the lack of information or the precision with which reality is translated into a mathematical formula. Such models cannot always wholly imitate real-world phenomena. As a result, its utilization is vital to humankind’s prophecy, which helps people comprehend what could ensue shortly to take preventative actions to avert worst-case scenarios. Compared to standard integer-order models, fractional-order (FO) models give a more precise and comprehensive insight into the complicated behavior of many diseases. FO systems are preferable to integer-order systems owing to their inherited features and memory description [11–13, 43, 44]. In addition, standard integer-order systems cannot investigate the dynamics between two points. Many ideas and conceptions about FO derivatives have been offered in the literature. The classical FO derivative is shown in [50] as an example. The FO, as mentioned earlier, derivatives [43, 44, 50] were effectively employed to represent real-world processes in various domains, including biology, engineering, and physics [12, 53]. Due to the non-singular kernel of the classical FO derivative, the non-local dynamics and crossover behavior of many real-world events cannot be adequately explained. In 2016, Atangana and Baleanu [48] presented a new FO derivative based on a generalized Mittag–Leffler function as a non-local and non-singular kernel to address some issues about the non-locality of the kernel of the derivative proposed in [51] and better to investigate the non-local complex behavior of various systems. The recently developed Atangana–Baleanu derivative [48] has been used to mimic a variety of real-world issues in diverse fields [52–55].

**Fig. 1:**
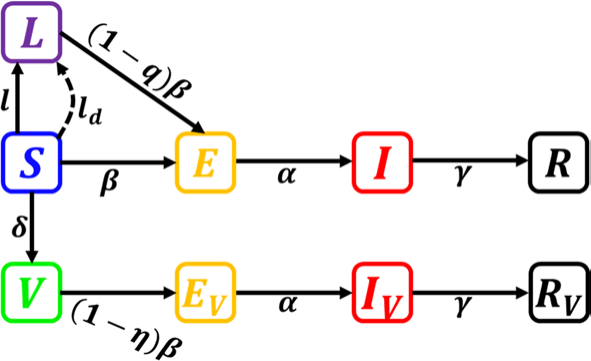
Schematic diagram of the model in which the population is divided into nine states: Susceptible $S(t)$, Lockdown $L(t)$, Exposed $E(t)$, Infected $I(t)$, Recovered $R(t)$, Vaccinated $V(t)$, Vaccinated exposed ${E}_{V}(t)$, Vaccinated infected ${I}_{V}(t)$ and Vaccinated Recovered ${R}_{V}\left(t\right)$

Initially, a few definitions relating to the classical Caputo [51] and Atangana–Baleanu fractional derivatives [48] are presented briefly (Fig. 1).

#### Definition 1

Under the condition of $F\left( t \right) \in {\mathcal{H}}^{1} \left( {0,T} \right),$ the general definition of ${{\mathbb{A}}{\mathbb{B}}{\mathbb{C}}}$ derivative of a function $F\left( t \right)$ is as follows:

7.1 $${}_{0}^{{{{\mathbb{A}}{\mathbb{B}}{\mathbb{C}}}}} D_{t}^{\alpha } F\left( t \right) = \frac{{{{\mathbb{A}}{\mathbb{B}}{\mathbb{C}}}\left( \alpha \right)}}{1 - \alpha }\mathop \smallint \limits_{0}^{t} \frac{{\text{d}}}{{{\text{d}}v}}F\left( t \right)\varepsilon_{\alpha } \left[ {\frac{ - \alpha }{{1 - \alpha }}\left( {t - v} \right)^{\alpha } } \right]{\text{d}}v.$$

In Eq. (7.1), substituting $\varepsilon_{\alpha } \left[ {\frac{ - \alpha }{{1 - \alpha }}\left( {t - v} \right)^{\alpha } } \right]$ by $\varepsilon_{1} = \exp \left[ {\frac{ - \alpha }{{1 - \alpha }}\left( {t - v} \right)} \right]$ for the Capto–Fabrizo differential operator. On top of that, it is to be mentionable that ${}_{0}^{{{{\mathbb{A}}{\mathbb{B}}{\mathbb{C}}}}} D_{t}^{\alpha } \left[ {{\text{constant}}} \right] = 0.$

The normalization function is denoted by the symbol ${{\mathbb{A}}{\mathbb{B}}{\mathbb{C}}}\left( \alpha \right)$, and its definition is as follows: ${{\mathbb{A}}{\mathbb{B}}{\mathbb{C}}}\left( 0 \right) = {{\mathbb{A}}{\mathbb{B}}{\mathbb{C}}}\left( 1 \right) = 1.$ Additionally, $\varepsilon_{\alpha }$ represents a unique function that is referred to as the Mittag–Leffler function.

#### Definition 2

If we assume that $F\left( t \right)$ is a function of the interval $L\left[ {0, T} \right]$, then the integral that corresponds to it in the ${{\mathbb{A}}{\mathbb{B}}{\mathbb{C}}}$ sense is provided by:

7.2 $${}_{0}^{{{{\mathbb{A}}{\mathbb{B}}{\mathbb{C}}}}} {\varvec{I}}_{t}^{\alpha } F\left( t \right) = \frac{1 - \alpha }{{{{\mathbb{A}}{\mathbb{B}}}{\mathbb{C}}\left( \alpha \right)}}F\left( t \right) + \frac{\alpha }{{{{\mathbb{A}}{\mathbb{B}}{\mathbb{C}}}\left( \alpha \right){\Gamma }\left( \alpha \right)}}\mathop \smallint \limits_{0}^{t} \left( {t - v} \right)^{\alpha - 1} F\left( v \right){\text{d}}v.$$

#### Lemma 1

According to proposition 3, described in [56], the desired solution of the assumed problem for the fractional order $0 < \alpha \le 1$ is.

$${}_{0}^{{{{\mathbb{A}}{\mathbb{B}}{\mathbb{C}}}}} {\varvec{D}}_{t}^{\alpha } F\left( t \right) = y\left( t \right), t \in \left[ {0,T} \right],$$

$$F\left( 0 \right) = F_{0} .$$

Considering that the right side disappears at time $t = 0$, then

7.3 $$F\left( t \right) = F_{0} + \frac{1 - \alpha }{{{{\mathbb{A}}{\mathbb{B}}{\mathbb{C}}}\left( \alpha \right)}}y\left( t \right) + \frac{\alpha }{{{\Gamma }\left( \alpha \right){{\mathbb{A}}{\mathbb{B}}{\mathbb{C}}}\left( \alpha \right)}}\mathop \smallint \limits_{0}^{t} \left( {t - v} \right)^{\alpha - 1} y\left( v \right){\text{d}}v.$$

### ABC fractional-order model

The system of the nonlinear ABC fractional-order differential equation (FODE) is thus as follows:

8 $$\begin{aligned} {}_{0}^{{ABC}} D_{t}^{\epsilon } S\left( t \right) & = - \beta S\left( t \right)\left( {I\left( t \right) + I_{V} \left( t \right)} \right) - \delta ~S\left( t \right) - lS\left( t \right) + l_{d} L\left( t \right), \\ {}_{0}^{{ABC}} D_{t}^{\epsilon } L\left( t \right) & = lS\left( t \right) - \left( {1 - q} \right)\beta L\left( t \right)\left( {I\left( t \right) + I_{V} \left( t \right)} \right) - l_{d} L\left( t \right), \\ {}_{0}^{{ABC}} D_{t}^{\epsilon } E\left( t \right) & = \beta S\left( t \right)\left( {I\left( t \right) + I_{V} \left( t \right)} \right) + \left( {1 - q} \right)\beta L\left( t \right)\left( {I\left( t \right) + I_{V} \left( t \right)} \right)~ - \alpha E\left( t \right), \\ {}_{0}^{{ABC}} D_{t}^{\epsilon } I\left( t \right) & = \alpha E\left( t \right) - \gamma I\left( t \right), \\ {}_{0}^{{ABC}} D_{t}^{\epsilon } R\left( t \right) & = \gamma I\left( t \right), \\ {}_{0}^{{ABC}} D_{t}^{\epsilon } V\left( t \right) & = \delta ~S\left( t \right) - \left( {1 - \eta } \right)\beta V\left( t \right)\left( {I\left( t \right) + I_{V} \left( t \right)} \right), \\ {}_{0}^{{ABC}} D_{t}^{\epsilon } E_{V} \left( t \right) & = \left( {1 - \eta } \right)\beta V\left( t \right)\left( {I\left( t \right) + I_{V} \left( t \right)} \right) - \alpha E_{V} \left( t \right), \\ {}_{0}^{{ABC}} D_{t}^{\epsilon } I_{V} \left( t \right) & = \alpha E_{V} \left( t \right) - \gamma I_{V} \left( t \right), \\ {}_{0}^{{ABC}} D_{t}^{\epsilon } R_{V} \left( t \right) & = \gamma I_{V} \left( t \right). \\ \end{aligned}$$

According to [51], the mathematical model (8) forecasts everyday features. Thus.

$$R_{ + }^{9} = \left\{ {\zeta \in R^{9} :\zeta \ge 0} \right\}$$ and

$$\zeta \left( t \right) = [S\left( t \right),L\left( t \right),E\left( t \right),I\left( t \right),R\left( t \right),V\left( t \right),E_{V} \left( t \right),I_{V} \left( t \right),R_{V} \left( t \right)]^{T} .$$

The method is organized and demonstrates mass conservation law; therefore, all Eqs. (8) are summated equal to zero. Directly,

$${}_{0}^{{ABC}} D_{t}^{\epsilon } \{ S\left( t \right) + L\left( t \right) + E\left( t \right) + I\left( t \right) + R\left( t \right) + V\left( t \right) + E_{V} \left( t \right) + I_{V} \left( t \right) + R_{V} \left( t \right) = 0,$$

indicates that the entire population remains stable.

Now, we may assume that $\mathop \sum \limits_{\forall i} \zeta \left( i \right) = 1$, where 1 signifies the whole population $N\left( t \right)$. Furthermore, all-state variables indicate entire population compartments.

#### Lemma 3.1

*The suggested model* (*Eq. *8) *solution* $\zeta \left( t \right)$ *is positive, unique, and lies in* $R_{ + }^{9}$*.*

#### Proof

All elements are kept in the positive quadrant to test the models’ positivity. As a result, the vector field tends towards $R_{ + }^{9}$, then.

9 $$\begin{aligned} {}_{0}^{{ABC}} D_{t}^{\epsilon } S\left( t \right) & = l_{d} L\left( t \right) \ge 0, \\ {}_{0}^{{ABC}} D_{t}^{\epsilon } L\left( t \right) & = 0, \\ {}_{0}^{{ABC}} D_{t}^{\epsilon } E\left( t \right) & = \beta S\left( t \right)\left( {I\left( t \right) + I_{V} \left( t \right)} \right) + \left( {1 - q} \right)\beta L\left( t \right)\left( {I\left( t \right) + I_{V} \left( t \right)} \right) \ge 0, \\ {}_{0}^{{ABC}} D_{t}^{\epsilon } I\left( t \right) & = \alpha E\left( t \right) \ge 0, \\ {}_{0}^{{ABC}} D_{t}^{\epsilon } R\left( t \right) & = \alpha I\left( t \right) \ge 0, \\ {}_{0}^{{ABC}} D_{t}^{\epsilon } V\left( t \right) & = \delta ~S\left( t \right) - \left( {1 - \eta } \right)\beta V\left( t \right)\left( {I\left( t \right) + I_{V} \left( t \right)} \right) \ge 0, \\ {}_{0}^{{ABC}} D_{t}^{\epsilon } E_{V} \left( t \right) & = \left( {1 - \eta } \right)\beta V\left( t \right)\left( {I\left( t \right) + I_{V} \left( t \right)} \right) \ge 0, \\ {}_{0}^{{ABC}} D_{t}^{\epsilon } I_{V} \left( t \right) & = \alpha E_{V} \left( t \right) \ge 0, \\ {}_{0}^{{ABC}} D_{t}^{\epsilon } R_{V} \left( t \right) & = \gamma I_{V} \left( t \right) \ge 0. \\ \end{aligned}$$

**Note:** Detailed theoretical analysis is in Appendix.

### Numerical analysis

In numerical analysis, finite-difference methods, network analysis, and fractional-order numerical schemes offer distinct approaches to epidemic modeling, each with significant differences. Finite-difference methods discretize space and time, describing the epidemic model dynamics, which are flexible, stable, and relatively simple to implement, making them suitable for various scenarios. On the other hand, network analysis focuses on representing populations as interconnected nodes, capturing realistic contact structures. This approach excels in modeling heterogeneous connectivity and dynamic interactions within complex networks, providing insights into the spread of diseases through communities. Fractional-order numerical schemes extend traditional calculus to include derivatives of non-integer order, enabling the modeling of memory effects and sub- or super-diffusive behaviors. This approach is advantageous when traditional models struggle to capture long-term dependencies in epidemic dynamics. In recapitulation, finite-difference methods are versatile and numerically stable, network analysis represents complex contact structures, and fractional-order schemes provide a more nuanced understanding of memory effects in epidemic modeling.

### Deterministic (ODE)

We have finally finished putting in place all of the necessary analytical structures, which allows us to use an explicit finite difference approach to solve the system of nonlinear Eq. (1) numerically; the results are presented and discussed below. We started with the assumption that $S(0)=0.9998,L(0)=0.0,E(0)=0.0,I(0)=0.0001,R(0)=0.0,V(0)=0.0, {E}_{V}(0)=0.0,{I}_{V}(0)=0.0001,{R}_{V}(0)=0.0$.

### Erdös and Rényi (ER) random network

The research conducted by Erdös and Rényi on random graphs, which involve the random connection of vertices, made a substantial contribution to establishing network science [33, 58]. Although distinct from preexisting networks, the random graph model plays a vital role in demonstrating the presence of graphs that satisfy specific criteria or elucidate nearly ubiquitous characteristics. The most straightforward approach to constructing a random graph involves establishing connections between potential pairs of vertices with a probability $p$, continuing this process until all pairs have been connected. The mathematical expression representing the number of edges in an Erdős-Rényi graph, characterized by $N$ nodes and a given probability $p$, can be formulated as follows:

10.1 $$E = N\left( {N - 1} \right)\frac{p}{2}.$$

where $N$ is the total number of nodes in the graph and $p = \frac{\left\langle k \right\rangle }{{N - 1}}$ is the probability that any two nodes will be connected by an edge. This equation holds because there are $\frac{{N\left( {N - 1} \right)}}{2}$ possible pairs of nodes in the graph and each pair of nodes is connected by an edge with probability $p.$

### Barabasi and Albert (BA) scale-free network

The mathematical model of network evolution featuring hubs and a scale-free degree distribution was proposed by Barabasi and Albert [34]. Additional nodes are introduced into an initial nucleus and are selectively connected to pre-existing nodes with more connections. The proposed model effectively incorporates the characteristics observed in various networks, encompassing domains such as social media, biology, physics, and computer science. The mathematical expression representing the number of edges in a Barabási–Albert network, given a total of $N$ nodes, is as follows:

10.2 $$E = m\left( {N - 1} \right),$$

where $m$ is the number of edges added to the graph at each time step during the initial growth phase of the network.

$$\left\langle k \right\rangle = 2m.$$

### Fractional-order (FO)

The numerical methodology for solving nonlinear fractional-order differential equations with fractional derivatives and non-local, non-singular kernels will be developed in this subsection. Now, we will look at the nonlinear fractional-order ordinary equation provided below to do this:

11.1 $$\left\{ {\begin{array}{*{20}l} {{}_{0}^{{{{\mathbb{A}}{\mathbb{B}}{\mathbb{C}}}}} D_{t}^{\varepsilon } x\left( t \right) = f\left( {t,x\left( t \right)} \right),} \hfill \\ {x\left( 0 \right) = x_{0} .} \hfill \\ \end{array} } \right.$$

By using the fundamental theorem of fractional calculus, Eq. (11.1) can be transformed into a fractional integral equation as follows:

11.2 $$x\left( t \right) - x\left( 0 \right) = \frac{1 - \varepsilon }{{{{\mathbb{A}}{\mathbb{B}}{\mathbb{C}}}\left( \varepsilon \right)}}f\left( {t,x\left( t \right)} \right) + \frac{\varepsilon }{{{\Gamma }\left( \varepsilon \right){{\mathbb{A}}{\mathbb{B}}{\mathbb{C}}}\left( \varepsilon \right)}}\mathop \smallint \limits_{0}^{t} \left( {t - v} \right)^{\varepsilon - 1} f\left( {v,x\left( v \right)} \right)dv.$$

One can be written Eq. (11.2) at the point $t_{n + 1} ,n = 0,1,2, \ldots$ as follows

11.3 $$\begin{aligned} x\left( {t_{n + 1} } \right) - x\left( 0 \right) & = \frac{1 - \varepsilon }{{{{\mathbb{A}}{\mathbb{B}}{\mathbb{C}}}\left( \varepsilon \right)}}f\left( {t_{n} ,x\left( {t_{n} } \right)} \right) + \frac{\varepsilon }{{{\Gamma }\left( \varepsilon \right){{\mathbb{A}}{\mathbb{B}}{\mathbb{C}}}\left( \varepsilon \right)}}\mathop \smallint \limits_{0}^{{t_{n + 1} }} \left( {t_{n + 1} - v} \right)^{\varepsilon - 1} f\left( {v,x\left( v \right)} \right)dv \\ & = \frac{1 - \varepsilon }{{{{\mathbb{A}}{\mathbb{B}}{\mathbb{C}}}\left( \varepsilon \right)}}f\left( {t_{n} ,x\left( {t_{n} } \right)} \right) + \frac{\varepsilon }{{{\Gamma }\left( \varepsilon \right){{\mathbb{A}}{\mathbb{B}}{\mathbb{C}}}\left( \varepsilon \right)}}\mathop \sum \limits_{k = 0}^{n} \mathop \smallint \limits_{{t_{k} }}^{{t_{k + 1} }} \left( {t_{k + 1} - v} \right)^{\varepsilon - 1} f\left( {v,x\left( v \right)} \right)dv. \\ \end{aligned}$$

Using a two-step Lagrange polynomial interpolation, one can estimate the function $f\left( {v,x\left( v \right)} \right),$ in the interval $\left[ {t_{k} ,t_{k + 1} } \right]$ as follows:

11.4 $$\begin{aligned} P_{k} \left( v \right) & = \frac{{v - t_{k - 1} }}{{t_{k} - t_{k - 1} }}f\left( {t_{k} ,x\left( {t_{k} } \right)} \right) + \frac{{v - t_{k} }}{{t_{k} - t_{k - 1} }}f\left( {t_{k - 1} ,x\left( {t_{k - 1} } \right)} \right) \\ & = \frac{{f\left( {t_{k} ,x\left( {t_{k} } \right)} \right)}}{h}\left( {v - t_{k - 1} } \right) + \frac{{f\left( {t_{k - 1} ,x\left( {t_{k - 1} } \right)} \right)}}{h}\left( {v - t_{k} } \right) \\ & \simeq \frac{{f\left( {t_{k} ,x_{k} } \right)}}{h}\left( {v - t_{k - 1} } \right) + \frac{{f\left( {t_{k - 1} ,x_{k - 1} } \right)}}{h}\left( {v - t_{k} } \right). \\ \end{aligned}$$

Thus, Eq. (11.3) can be written as

11.5 $$\begin{aligned} x_{n + 1} & = x_{0} + \frac{1 - \varepsilon }{{{{\mathbb{A}}{\mathbb{B}}{\mathbb{C}}}\left( \varepsilon \right)}}f\left( {t_{n} ,x\left( {t_{n} } \right)} \right) \\ & \quad + \frac{\varepsilon }{{{\Gamma }\left( \varepsilon \right){{\mathbb{A}}{\mathbb{B}}{\mathbb{C}}}\left( \varepsilon \right)}}\mathop \sum \limits_{k = 0}^{n} \left( {\frac{{f\left( {t_{k} ,x_{k} } \right)}}{h}\mathop \smallint \limits_{{t_{k} }}^{{t_{k + 1} }} \left( {v - t_{k - 1} } \right)\left( {t_{n + 1} - v} \right)^{\varepsilon - 1} {\text{d}}v } \right. \\ & \quad \left. { - \frac{{f\left( {t_{k - 1} ,x_{k - 1} } \right)}}{h}\mathop \smallint \limits_{{t_{k} }}^{{t_{k + 1} }} \left( {v - t_{k} } \right)\left( {t_{n + 1} - v} \right)^{\varepsilon - 1} {\text{d}}v } \right). \\ \end{aligned}$$

Let us assume,

11.6 $$\begin{aligned} & \mathop \smallint \limits_{{t_{k} }}^{{t_{k + 1} }} \left( {v - t_{k - 1} } \right)\left( {t_{n + 1} - v} \right)^{\varepsilon - 1} {\text{d}}v = A_{\varepsilon ,k,1} , \\ & \mathop \smallint \limits_{{t_{k} }}^{{t_{k + 1} }} \left( {v - t_{k} } \right)\left( {t_{n + 1} - v} \right)^{\varepsilon - 1} {\text{d}}v = A_{\varepsilon ,k,2} . \\ \end{aligned}$$

Then, we have

11.7 $$\begin{aligned} A_{\varepsilon ,k,1} & = h^{\varepsilon + 1} \frac{{\left( {n + 1 - k} \right)^{\varepsilon } \left( {n - k + 2 + \varepsilon } \right) - \left( {n - k} \right)^{\varepsilon } \left( {n - k + 2 + 2\varepsilon } \right)}}{{\varepsilon \left( {\varepsilon + 1} \right)}}, \\ A_{\varepsilon ,k,2} & = h^{\varepsilon + 1} \frac{{\left( {n + 1 - k} \right)^{\varepsilon + 1} - \left( {n - k} \right)^{\varepsilon } \left( {n - k + 1 + \varepsilon } \right)}}{{\varepsilon \left( {\varepsilon + 1} \right)}}. \\ \end{aligned}$$

Substituting the value of Eqs. (11.6) and (11.7) in Eq. (11.5), we have

11.8 $$\begin{aligned} x_{n + 1} & = x_{0} + \frac{1 - \varepsilon }{{{{\mathbb{A}}{\mathbb{B}}{\mathbb{C}}}\left( \varepsilon \right)}}f\left( {t_{n} ,x\left( {t_{n} } \right)} \right) \\ & \quad + \frac{{\varepsilon h^{\varepsilon } }}{{{\Gamma }\left( {\varepsilon + 2} \right){{\mathbb{A}}{\mathbb{B}}{\mathbb{C}}}\left( \varepsilon \right)}}\mathop \sum \limits_{k = 0}^{n} \left( {f\left( {t_{k} ,x_{k} } \right)\left( {\left( {n + 1 - k} \right)^{\varepsilon } \left( {n - k + 2 + \varepsilon } \right)} \right.} \right. \\ & \quad \left. { - \left( {n - k} \right)^{\varepsilon } \left( {n - k + 2 + 2\varepsilon } \right)} \right) - f\left( {t_{k - 1} ,x_{k - 1} } \right)\left( {\left( {n + 1 - k} \right)^{\varepsilon + 1} - \left( {n - k} \right)^{\varepsilon } \left( n \right.} \right. \\ & \quad - \left. {\left. {\left( {k + 1 + \varepsilon } \right)} \right)} \right) \\ \end{aligned}$$

Then, for susceptible human compartments, one can write,

11.9 $$\begin{aligned} S_{n + 1} & = S_{0} + \frac{1 - \varepsilon }{{{{\mathbb{A}}{\mathbb{B}}{\mathbb{C}}}\left( \varepsilon \right)}}f\left( {t_{n} ,S\left( {t_{n} } \right)} \right) \\ & \quad + \frac{{\varepsilon h^{\varepsilon } }}{{{\Gamma }\left( {\varepsilon + 2} \right){{\mathbb{A}}{\mathbb{B}}{\mathbb{C}}}\left( \varepsilon \right)}}\mathop \sum \limits_{k = 0}^{n} \left( {f\left( {t_{k} ,S_{k} } \right)\left( {\left( {n + 1 - k} \right)^{\varepsilon } \left( {n - k + 2 + \varepsilon } \right)} \right.} \right. \\ & \quad \left. { - \left( {n - k} \right)^{\varepsilon } \left( {n - k + 2 + 2\varepsilon } \right)} \right) - f\left( {t_{k - 1} ,S_{k - 1} } \right)(\left( {n + 1 - k} \right)^{\varepsilon + 1} - \left( {n - k} \right)^{\varepsilon } \left( n \right. \\ & \quad \left. {\left. {\left. { - k + 1 + \varepsilon } \right)} \right)} \right) \\ \end{aligned}$$

The remaining compartment’s equations are the same as above.

## Results and discussion

According to [49], the proposed model is biologically significant, and its solution is positive for all $t\ge 0$*.* On top of that, specified by the entire population $N\left(t\right)$ [12]. In accordance, we prove that the proposed model is globally stable. On top of that, we deduce the point of equilibrium, which is ${\varepsilon }_{0}=$(0, 0, 0, 0, 0, 0, 0, 0, 0). Also, discuss the basic and effective reproduction number (see Appendix) and their relationship, the relationship between critical vaccine proportion, which demonstrates the real scenarios of vaccine efficacy rate and basic reproduction number’s value. If policymakers in different countries maintain the proposed model’s strategy, they can control the epidemic without making any waves. In addition, we also analyzed the LF’s first and second derivatives. The second derivative of the LF informs us of the curvature based on its sign, whereas the first derivative tells us about the progression of the disease. Furthermore, the clarification of the proposed system of nonlinear equations is unique according to [12, 57] (see Appendix).

**Fig. 2:**
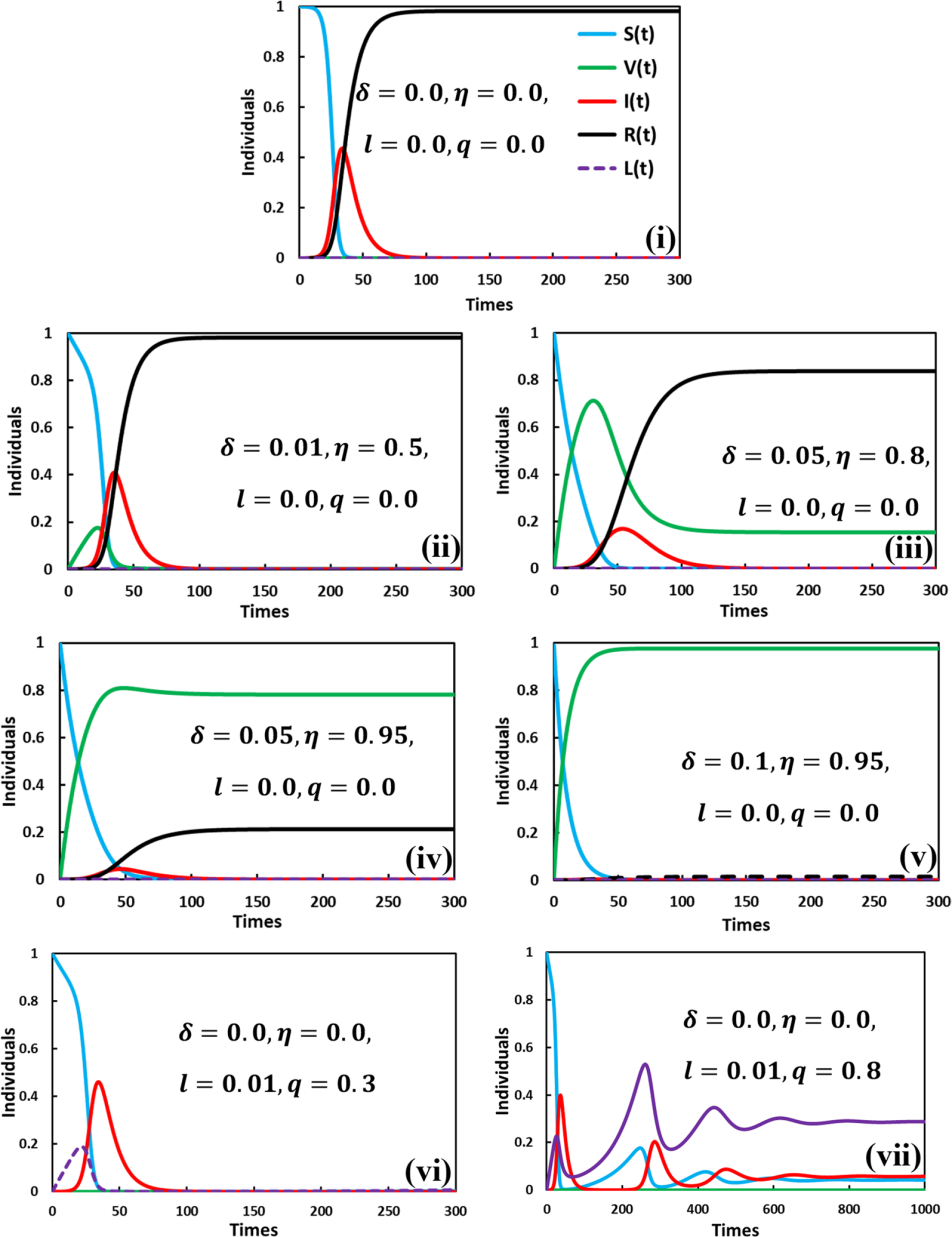
Final epidemic size $(R\left(\infty \right))$ colored with black, suspected susceptible $(S\left(\infty \right))$ colored with blue, lockdown $(L\left(\infty \right))$ colored with violet, infected $(I\left(\infty \right))$ colored with red and vaccinated $\left(V\left(\infty \right)\right)$ with green. Parameters used are **i** $\beta =1.0, \gamma =0.1, \alpha =1/5,\eta =0.0, q=0.0, l=0.0$ and $\delta =0.0$. **ii** $\beta =1.0, \gamma =0.1, \alpha =1/5,\eta =0.5, q=0.0, l=0.0$ and $\delta =0.01$. **iii** $\beta =1.0, \gamma =0.1, \alpha =1/5,\eta =0.8, q=0.0, l=0.0$ and $\delta =0.05$. **iv** $\beta =1.0, \gamma =0.1, \alpha =1/5,\eta =0.95, q=0.0, l=0.0$ and $\delta =0.05.$ **v** $\beta =1.0, \gamma =0.1, \alpha =1/5,\eta =0.95, q=0.0, l=0.0$ and $\delta =0.1$. **vi** $\beta =1.0, \gamma =0.1, \alpha =1/5,\eta =0.0, q=0.3, l=0.01$ and $\delta =0.0$. and (vii) $\beta =1.0, \gamma =0.1, \alpha =1/5,\eta =0.0, q=0.8, l=0.01$ and $\delta =0.0$

Initially, we focused on the result of numerical simulation for the time-evolving curve about the endemic steadiness of the proposed model. The time series of susceptible, vaccinated, infected, lockdown, and recovered individuals are portrayed in Fig. 2, which presents the changing behavior of the controlling parameters $\delta , \eta , l,$ and $q$, respectively. The baseline system values are defined as the default case $(\beta =1.0, \gamma =0.1, \alpha =1/5)$ presented in Fig. 2i. Due to lower vaccine effectiveness and vaccination rate (Fig. 2ii), the disease incidence shows a similar tendency as in the default case; the vaccine does not work. However, Fig. 2iii reveals that increasing the vaccination rate and vaccine effectiveness reduced the pick of infected individuals and the final epidemic size (recovered). Interestingly, the vaccinated emerges at a sporadic peak before stabilizing at equilibrium. As time passes, some people cannot maintain their health due to the vaccine’s ineffectiveness (50 percent are perfectly immune, and the remaining are non-immune).

Consequently, with the higher rate of the vaccination program and the high efficacy rate, the vaccine can control or eradicate the disease (see Fig. 2iv and v). Besides, suppose the vaccine is not available. In that case, the policymakers of different countries need to require alternate policies to control the diseases, such as lockdown, shutdown, state of emergency, and mask-wearing, that can help stop the spread of the COVID-19 virus. To represent the impact of lockdown irrespective of vaccination, we display Fig. 2vi and vii for the settings $q=0.3$ and $q=0.8$ (where $l=0.01$ and $\delta =\eta =0$). As shown in Fig. 2vi, the infected individuals remain unchanged with the lower lockdown maintain factor $q(=0.3)$. However, the number of infected individuals decreased with increasing $q$(Fig. 2vii). Thus, when the lockdown works properly (higher $q$), people are more compliant with maintaining the lockdown, which reduces the infected number of individuals. One interesting phenomenon of multi-wave characteristics observed in Fig. 2vii may be directed by the lockdown strategy.

**Fig. 3:**
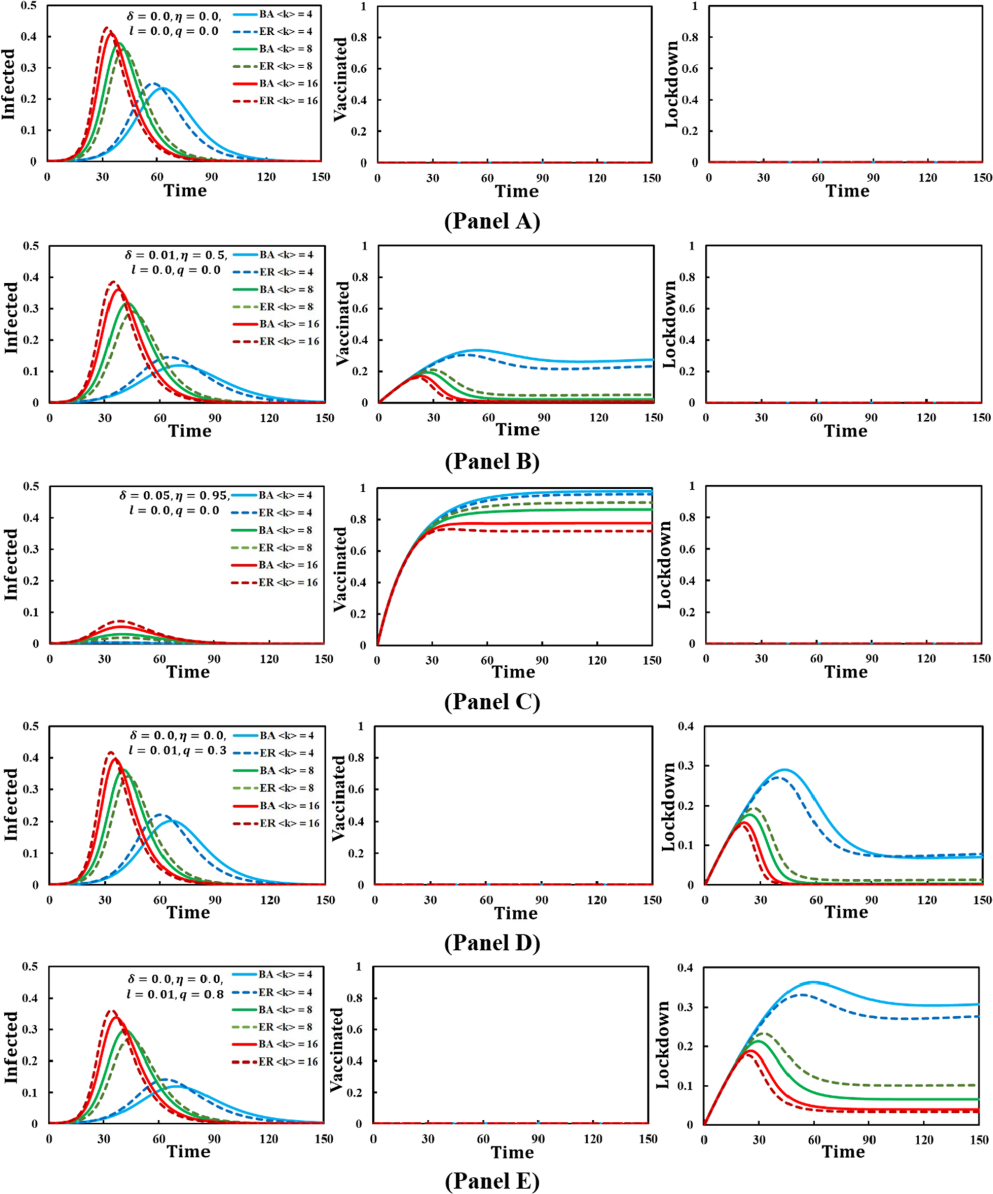
Representation of the disease dynamics infected, vaccinated, and lockdown state individuals in Erdös and Rényi (ER) random and Barabasi and Albert (BA) scale-free physical networks for average degree $<k>=\mathrm{4,8},16$. The parameter setting in panel **A** $\delta =0.0,\eta =0.0,l=0.0,q=0.0$, **B** $\delta =0.01,\eta =0.5,l=0.0,q=0.0$, **C** $\delta =0.05,\eta =0.95,l=0.0,q=0.0$, **D** $\delta =0.0,\eta =0.0,l=0.01,q=0.3$, **E** $\delta =0.0,\eta =0.0,l=0.01,q=0.8$ and the remaining parameters value are $\beta =1.0,\alpha =0.2,\gamma =0.1$

In Fig. 3, we conducted a study to examine the impact of disease diffusion using two spatial structures: ER-RG (Poisson) and BA-SF (power) networks. We considered five cases with average degree distributions of $<k> = 4, 8,$ and $16$.

**Table:** 

Cases	Settings
Panel A	$\delta =0.0; \eta =0.0; l=0.0; q=0.0$
Panel B	$\delta =0.01; \eta =0.5; l=0.0; q=0.0$
Panel C	$\delta =0.05; \eta =0.95; l=0.0; q=0.0$
Panel D	$\delta =0.0; \eta =0.0; l=0.01; q=0.3$
Panel E	$\delta =0.0; \eta =0.0; l=0.01; q=0.8$

Upon comparing the results of these cases, we observed a significant reduction in infection rates for Panel C when using $\delta =0.05$ and $\eta =0.95$. Conversely, Panel A experienced the highest number of infections when no provisions were considered, leading to maximum disease spread. Furthermore, in Panel E, we noticed that higher q values resulted in increased lockdown measures but also contributed to lower infection rates. In our investigation of the different settings involving average degree distributions $<k>$ and the two network types (ER-RG and BA-SF), we found that ER-RG showed the highest infection rate for $<k> = 16$ in Panel A. BA-SF exhibited a similar tendency but with slightly lower infection rates for $<k> = 16$. However, the infection rates were significantly lower when $<k>$ was set to 4 for ER-RG and BA-SF, which suggests that reducing the average degree in a network (i.e., decreasing the number of connections between individuals) leads to a decrease in the infection rate. Conversely, higher average degrees result in faster disease spread since individuals are more interconnected.

In Panels B and C, we observed that the implementation of vaccination led to higher vaccination rates in scenarios where the average degree distribution was lower. This trend can be explained by the fact that individuals exhibited reduced interaction rates as the average degree decreased. As a result, the average connectivity among individuals in the community decreased, leading to the emergence of social distancing practices. This combination of reduced connectivity and social distancing contributed to decreased infection rates and increased the number of vaccinated individuals. Consequently, the scenario with an average degree of $<k>=4$ resulted in the fewest individuals being infected, accompanied by higher vaccination rates. A similar pattern was observed in Panels D and E when the lockdown strategy was implemented. Lower average degree distributions were associated with a more effective lockdown effect. In such cases, lower connectivity and lockdown measures further facilitated social distancing and reduced disease transmission. Thus, the lower average degree distributions promote higher vaccination rates and more effective lockdown strategies due to the reduced connectivity among individuals, which leads to adopting social distancing practices and results in lower infection rates.

**Fig. 4:**
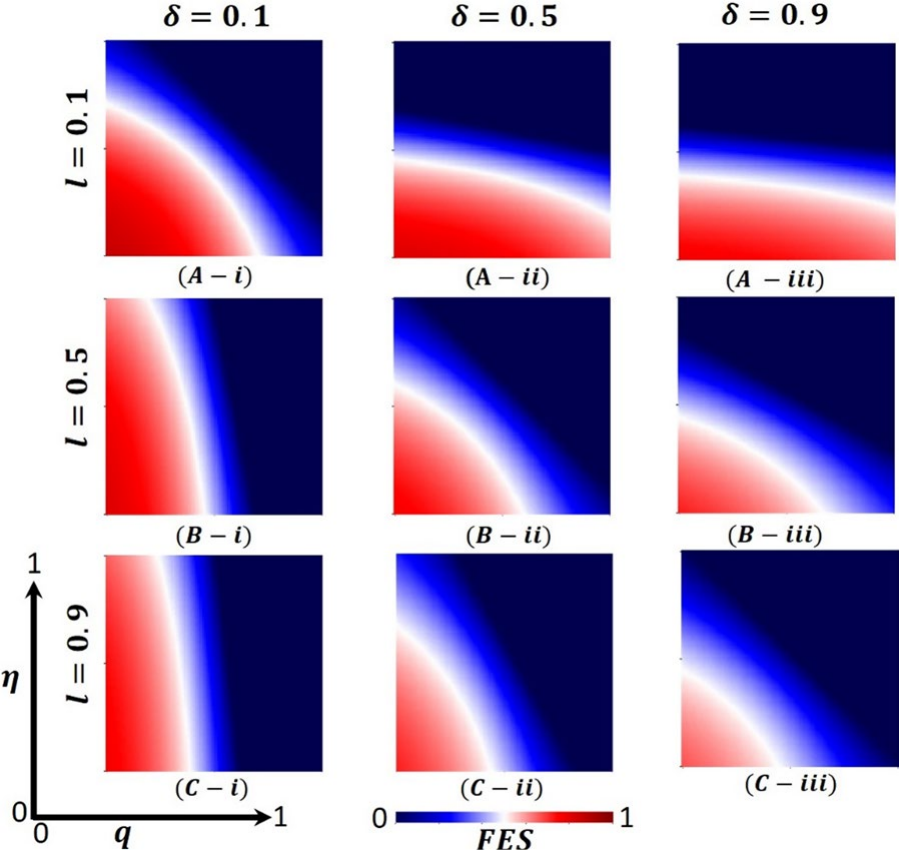
Presented is the final epidemic size (FES) for vaccine effectiveness $(\eta )$ and lockdown maintenance factor $(q)$. Subpanels (A-*), (B-*), and (C-*) show for the lockdown level $(l)$, whereas panels (*-i), (*-ii), (*-iii) vaccination rate $(\delta )$, respectively. Parameters used are $\beta =1.0, \gamma =0.1, \alpha =1/5, l=0.1, 0.5, 0.9$ and $\delta =0.1, 0.5, 0.9$

**Fig. 5:**
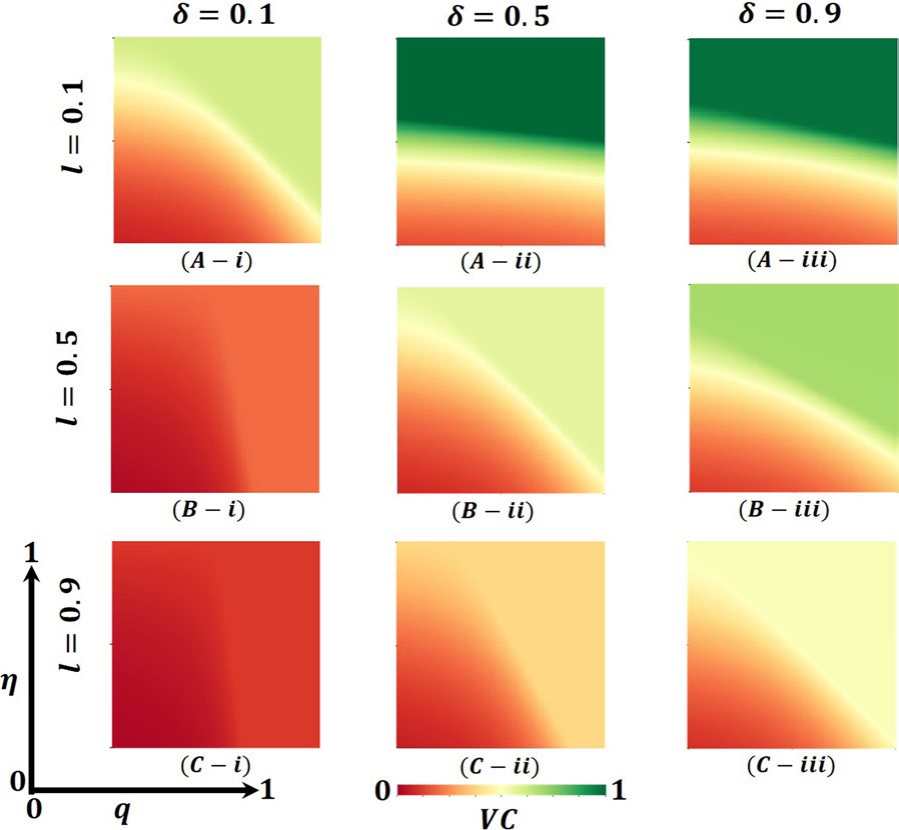
Presented is the vaccination coverage (VC) for vaccine effectiveness $(\eta )$ and lockdown maintenance factor $(q)$. Subpanels (A-*), (B-*), and (C-*) show the lockdown level $(l)$, whereas panels (*-i), (*-ii), (*-iii) vaccination rate $(\delta )$, respectively. Parameters used are $\beta =1.0, \gamma =0.1, \alpha =1/5, l=0.1, 0.5, 0.9$ and $\delta =0.1, 0.5, 0.9$

**Fig. 6:**
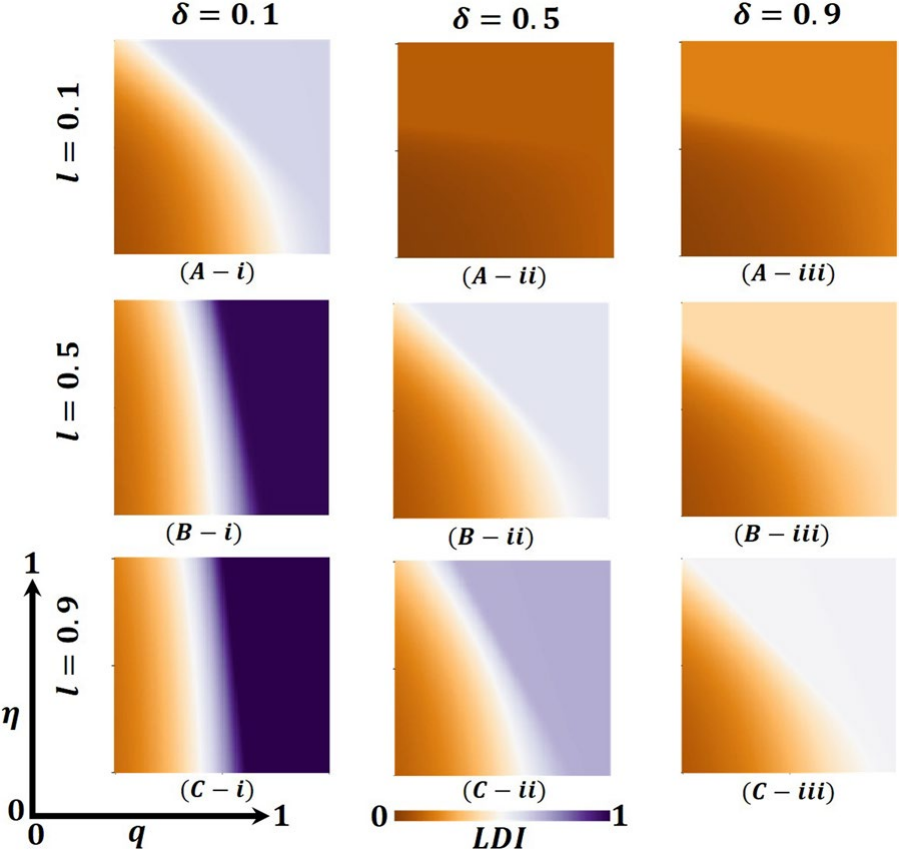
Presented is the lockdown Individuals (LDI) for vaccine effectiveness $(\eta )$ and lockdown maintenance factor $(q)$. Subpanels (A-*), (B-*), and (C-*) show the lockdown level $(l)$, whereas panels (*-i), (*-ii), (*-iii) vaccination rate $(\delta )$, respectively. Parameters used are $\beta =1.0, \gamma =0.1, \alpha =1/5, l=0.1, 0.5, 0.9$ and $\delta =0.1, 0.5, 0.9$

Overall, Figs. 4, 5, and 6 display the 2D heat maps of the vaccine efficacy rate ($\eta$) (x-axis) versus lockdown maintenance factor ($q$) (*y*-axis), which illustrate the final epidemic size (FES), vaccination coverage (VC) and Lockdown Individuals (LDI), respectively, of the epidemic at the equilibrium point $t\to \infty$. Moreover, panels (A-*), (B-*), and (C-*) show the results under the lockdown rate $l=0.1, 0.5,$ and $0.9$, respectively, whereas panels (*-i), (*-ii), and (*-iii) present the results for vaccination rate $\delta =0.1, 0.5$ and $0.9$, respectively. Each panel in Fig. 4 is partitioned into two equilibrium states: disease-free equilibrium (blue) and endemic equilibrium (deep red). As expected, reducing vaccine efficacy and lockdown maintenance factors increased the FES; the disease spread quickly. Nevertheless, the opposite tendency was found for higher $\eta$ and $q$ values.

In Fig. 4, panel (A-*) for the fixed lockdown level $l=0.1$ and increasing vaccination program $\delta =0.1, 0.5, 0.9$ gradually decreases the FES, as expected. The shape of every heat map changed from oblique to almost parallel. However, the indisputable fact is that as the lockdown level is low, people do not stay at home; as a result, the disease spreads to the whole society even if the vaccination program is increased, a real-world phenomenon. On top of that, in comparison with Fig. 4 with Figs. 5 and 6, panel (A-*), we see that in Fig. 5, most of the people are covered with vaccination (high green), whereas in Fig. 6, no people are in the lockdown provision (higher gray). Thus, if the lockdown level is low, but the vaccination program is increased (no vaccine shortage) with a higher efficacy rate, the disease is eradicated from society. In this context, the lockdown did not work enough. Again, for the fixed vaccination program $\delta =0.1$ and improving the $\mathrm{lockdown level }l=0.1, 0.5, 0.9,$ Fig. 4, in panels (A(i), B(i), C(i)), we see that FES slowly decreases, which is also practical. For example, countries like Japan and Bangladesh enhanced lower to higher lockdown levels and controlled the COVID-19 pandemic [59, 60]. In comparison with Fig. 4 with Figs. 5 and 6, panels (A(i), B(i), and C(i)), we see that in Fig. 5, vaccination did not work; a smaller number of the total population participates in the vaccination program. In contrast, most people are covered with the lockdown level (high violet), illustrated in Fig. 6. Therefore, if a vaccination program is not high enough, i.e., a vaccine shortage exists in a society with a high efficacy rate, higher lockdown levels with high maintenance factors help policymakers control the COVID-19 pandemic. For example, around 60% of the total population of the USA did not agree to take any vaccine and decided to maintain the lockdown policy [61]. However, according to Fig. 2, a non-pharmaceutical intervention lockdown is not a permanent solution; it has economic issues. It is a one-seasonal solution because those under lockdown after a certain period become susceptible again, confirming that a highly accurate vaccination program is a permanent solution for controlling any epidemic.

Figure 4, panel (B-*) illustrates that the increasing value of lockdown level $l=0.5$ and vaccination program $\delta =0.1, 0.5, 0.9$ decreases the FES quicker than in Fig. 4, panel (A-*). Every heat map’s size of the red region area unceasingly becomes smaller. When the lockdown level is medium with good maintenance and enhancing high efficacy vaccination programs, most people stay at home and are progressively vaccinated; the disease does not spread in society more quickly. Realistically, many countries (like Bangladesh) policymakers follow this strategy step by step to open essential offices/sectors, which also helps reduce economic loss [59]. Furthermore, panels (B-*) of Figs. 5 and 6 justify the illustration of Fig. 4, panel (B-*), that many people participate in the vaccination program and come out from the lockdown provision day by day to fulfill their daily needs. On the other hand, we see that FES reduces significantly for the vaccination program δ = 0.5 and the lockdown level $l=0.1, 0.5, 0.9$, Fig. 4, in panels (A(ii), B(ii), C(ii)) as expected. In contrast, Figs. 5 and 6 elucidate that vaccination coverage (VC) reduces and the number of lockdown individuals (LDI) increases. Thus, the medium-level lockdown policy with a gradual vaccination program aids in controlling an epidemic.

Furthermore, when the lockdown level is high (shutdown, state of emergency) $l= 0.9$ and maintenance, people who have locked their residences do not move elsewhere. In that case, increasing the rate of mass vaccination programs $\delta =0.1, 0.5, 0.9$ with a high efficacy rate considerably diminishes the FES of an epidemic (presented in Fig. 4, panel C-*) compared to panel B-*, C-*, which assists policymakers in managing worst situations of the country. The government of India has overcome such a situation by applying this policy [62]. Moreover, Fig. 5 reveals that in comparison with Fig. 4, the VC portion reduces more than panel B-*, as expected. When any region has a higher level of lockdown with maintenance and an increasing high-efficacy vaccination program, people assume that the epidemic has died out. As stated earlier, the multi-waving phenomenon arises in Fig. 2vii. On the other hand, Fig. 6 demonstrates that the number under the lockdown provision was reduced due to the increasing rate of the high-efficacy vaccination program. Again, more elevated level vaccination program $\delta =0.9$ with efficacy, and for the $\mathrm{lockdown level }l=0.1, 0.5, 0.9,$ Fig. 4, in panels (A(iii), B(iii), C(iii)), we see that FES turns into an endemic compared to panel (A-C(i-ii)). On top of that, the combined higher effect of the high efficacy vaccination program and increasing lockdown level eradication of the disease more quickly presented in Figs. 5 and 6, panels (A(iii), B(iii), C(iii)), validated the scenarios of Fig. 4, panel (A–C(iii)). Realistically, if 70% of the people participate in the vaccination program, the disease automatically becomes controlled, even dying out. However, it is not possible for poor, developing, and under-developing countries, but it is likely for rich countries because of economic facts and the availability of vaccines. Therefore, the combined effect of the high efficacy vaccination program and lockdown level very shortly assists policymakers in eradicating the disease from society.

Finally, suppose we concentrate our attention diagonally. In that case, the combined effect of the high efficacy of the available vaccination program and lockdown level significantly eradicated the FES quickly, as portrayed in Fig. 4. On top of that, Figs. 5 and 6 reveal that the combined effects work favorably. Moreover, it gives policymakers great hope in controlling the transmissible disease COVID-19 from society.

**Fig. 7:**
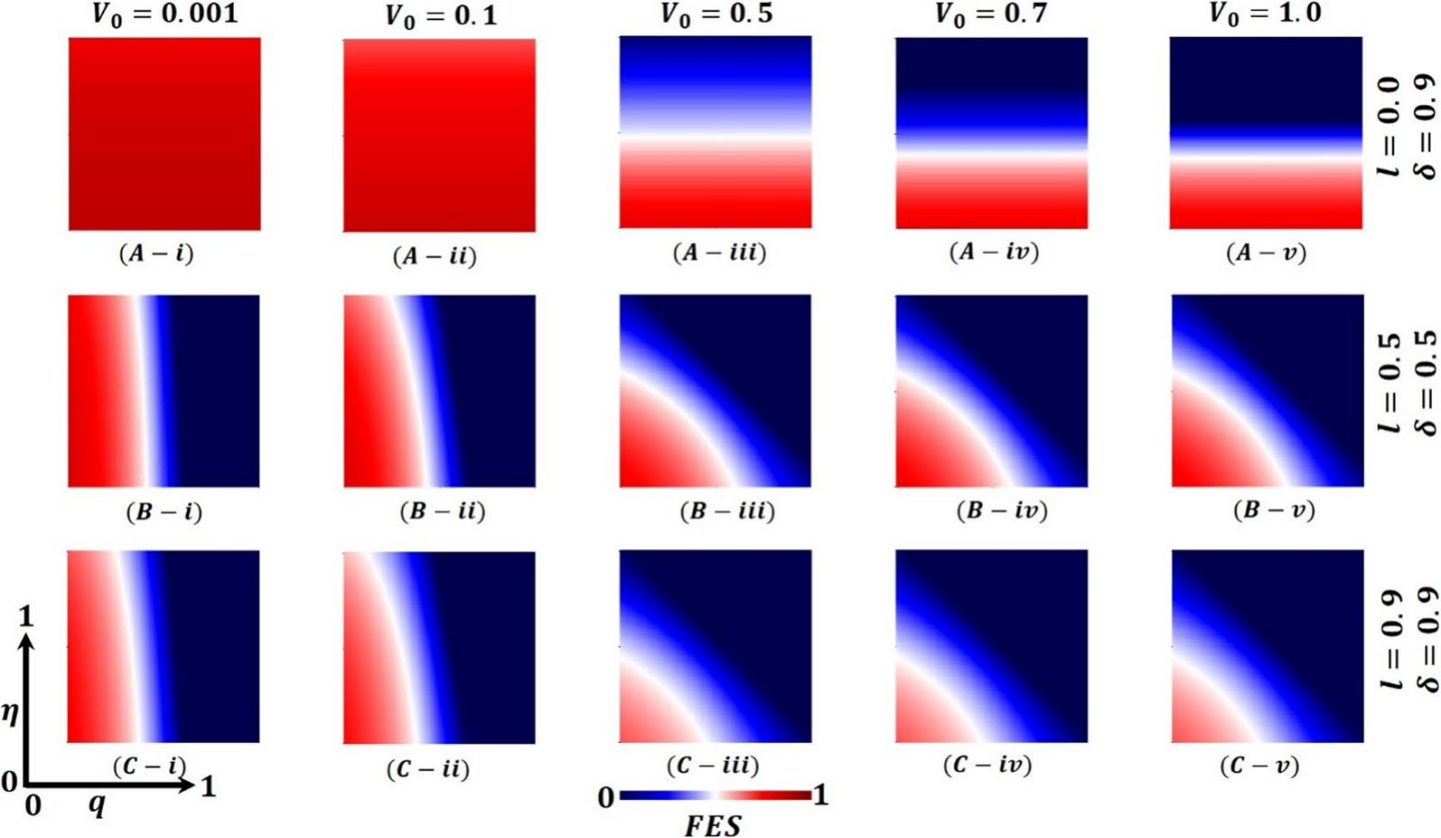
Presented is the final epidemic size (FES) for vaccine effectiveness $(\eta )$ and lockdown maintenance factor $(q)$. Subpanels (A-*), (B-*), and (C-*) show the results under the lockdown rate $l=0.0, 0.5, 0.9$ and vaccination rate $\delta =0.9, 0.5, 0.9$, respectively, whereas panels (*-i), (*-ii), (*-iii), (*-iv) and (*-v) present the results for vaccine availability rate ${V}_{0}=0.001, 0.1, 0.5, 0.7, 1.0, \beta =1.0, \gamma =0.1, {\text{and}} \alpha =1/5$, respectively

**Fig. 8:**
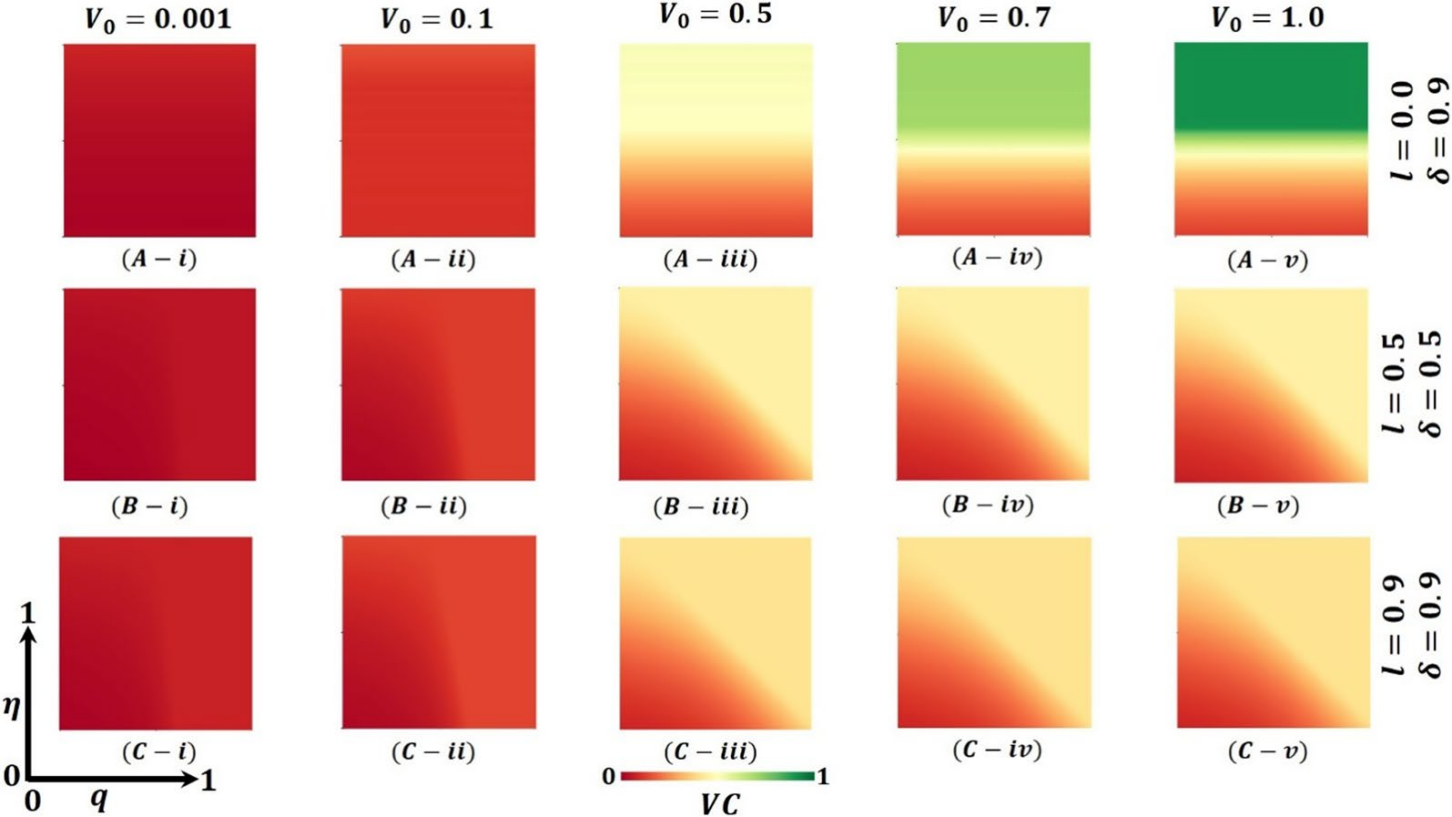
Presented is the vaccination coverage (VC) for vaccine effectiveness $(\eta )$ and lockdown maintenance factor $(q)$. Subpanels (A-*), (B-*), and (C-*) show the results under the lockdown rate $l=0.0, 0.5, 0.9$ and vaccination rate $\delta =0.9, 0.5, 0.9$, respectively, whereas panels (*-i), (*-ii), (*-iii), (*-iv) and (*-v) present the results for vaccine availability rate ${V}_{0}=0.001, 0.1, 0.5, 0.7, 1.0, \beta =1.0, \gamma =0.1,\mathrm{ and }\alpha =1/5$, respectively

**Fig. 9:**
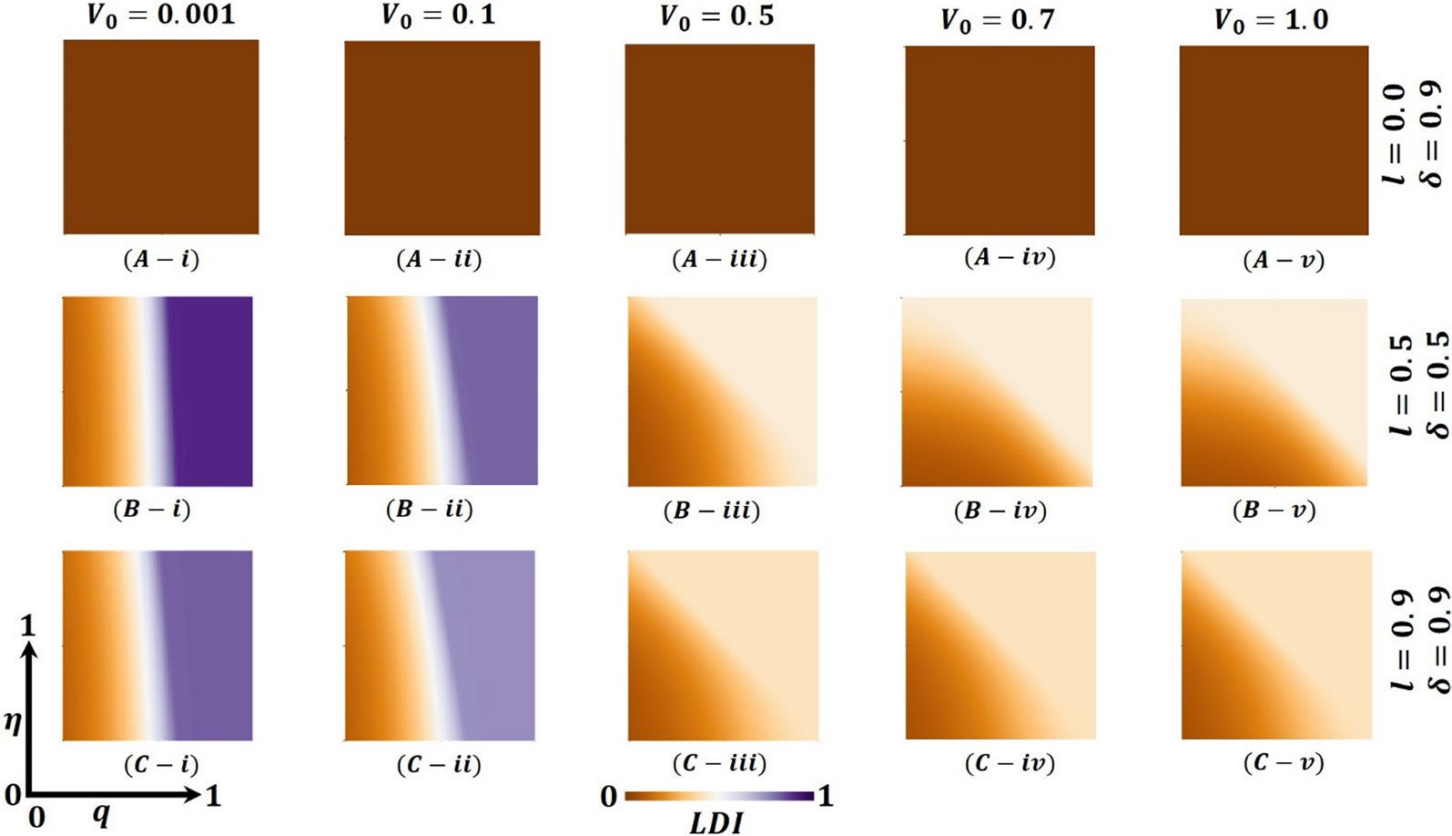
Presented the lockdown Individuals (LDI) for vaccine effectiveness $(\eta )$ and lockdown maintenance factor $(q)$. Subpanels (A-*), (B-*), and (C-*) show the results under the lockdown rate $l=0.0, 0.5, 0.9$ and vaccination rate $\delta =0.9, 0.5, 0.9$, respectively, whereas panels (*-i), (*-ii), (*-iii), (*-iv) and (*-v) present the results for vaccine availability rate ${V}_{0}=0.001, 0.1, 0.5, 0.7, 1.0, \beta =1.0, \gamma =0.1, {\text{and}} \alpha =1/5$, respectively

Further, Fig. 7 (FES), Fig. 8 (VC), and Fig. 9 (LDI) display the impact of vaccine shortage, vaccination rate, and lockdown effect as a form of 2D heat maps along with the vaccine efficacy rate ($\eta$) (*x*-axis) versus lockdown maintenance factor ($q$) (*y*-axis). Here, panels (A-*), (B-*), and (C-*) illustrate the outcomes under the settings $\left(l, \delta \right)=\left(0.0, 0.9\right),(\mathrm{0.5,0.5})$ and $(\mathrm{0.9,0.9})$ correspondingly, whereas panels (*-i), (*-ii), (*-iii), (*-iv) and (*-v) present the results for vaccine availability rate ${V}_{0}=0.001, 0.1, 0.5, 0.7,$ and $1.0$, respectively. Figure 7A-i and A-ii represents higher FES as the value of ${V}_{0}=0.001,$ and ${V}_{0}=0.1$ when $=0.0,\delta =0.9$, which resulted in a significant vaccine shortage. However, the FES reduced gradually as the rate of vaccine availability increased, displayed in Figs. 7A-iii–A-v (${V}_{0}=0.5, 0.7,$ and $1.0$). Furthermore, if the lockdown strategy is imposed with the vaccine program (Fig. 7B-*), it exhibits less FES than the no-lockdown policy. Consequently, Fig. 7C-* presents a reduced FES for full lockdown with a complete vaccine program. The country’s policymakers impose a 50% lockdown policy and continue 50% vaccination programs to control any epidemic disease when there is a vaccination shortage. Also, this strategy helps them to minimize economic loss. If the lockdown level is more stringent, $l=0.9$, and the vaccination rate is mass $\delta =0.9$, there is no shortage of vaccines, and soon, the pandemic may be eradicated. Finally, Figs. 8 and 9 show that vaccinated and lockdown individuals reduced the disease when the effectiveness/efficacy and acceptance rate were high. In practice, if 70% of the people participate in the vaccination program, the disease automatically becomes controlled, reflecting our current results.

Aside from conventional discussion, the proposed fractional-order model is also numerically simulated, and the results are displayed graphically. Let us first introduce the numerical method used to solve the model. [49] is utilized to carry out the numerical simulation to solve IVPs with ABC derivatives.

**Fig. 10:**
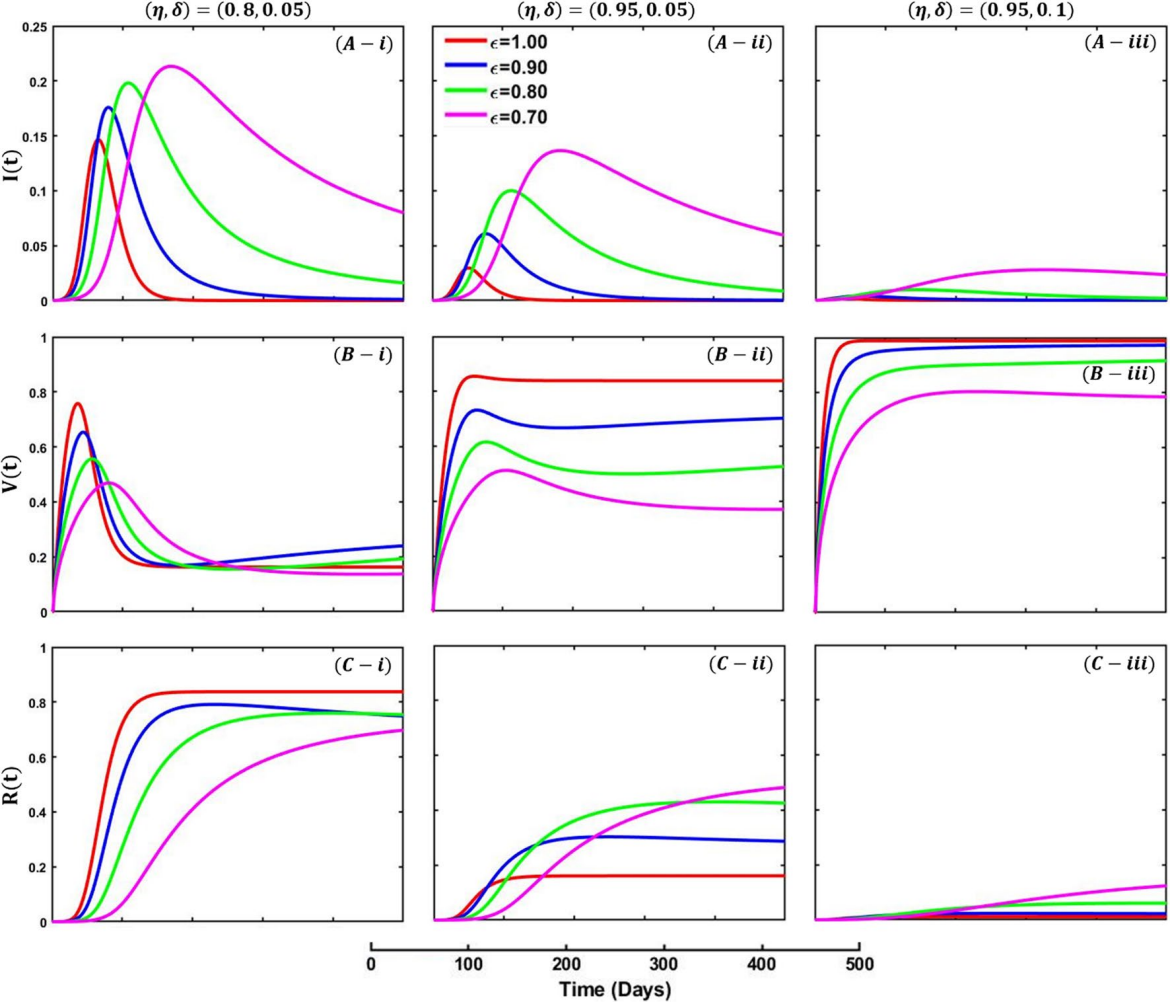
Presented is the effect of changing the fractional-order $\varepsilon =0.7, 0.8, 0.9, 1.0$ on the infected (Panel A-*), recovered (Panel B-*), and vaccinated (Panel C-*) individuals. Subpanels panel (*-i), panel (*-ii), and panel (*-iii) show the results under the vaccine efficacy rate and vaccination rate $\left(\eta ,\delta \right)=\left(\mathrm{0.8,0.05}\right), (\mathrm{0.95,0.05})$ and $(\mathrm{0.95,0.1})$, respectively, whereas the remaining parameter settings are $\beta =1.0, \gamma =0.1, \alpha =1/5, q=0.0,$ $l=0.0$

**Fig. 11:**
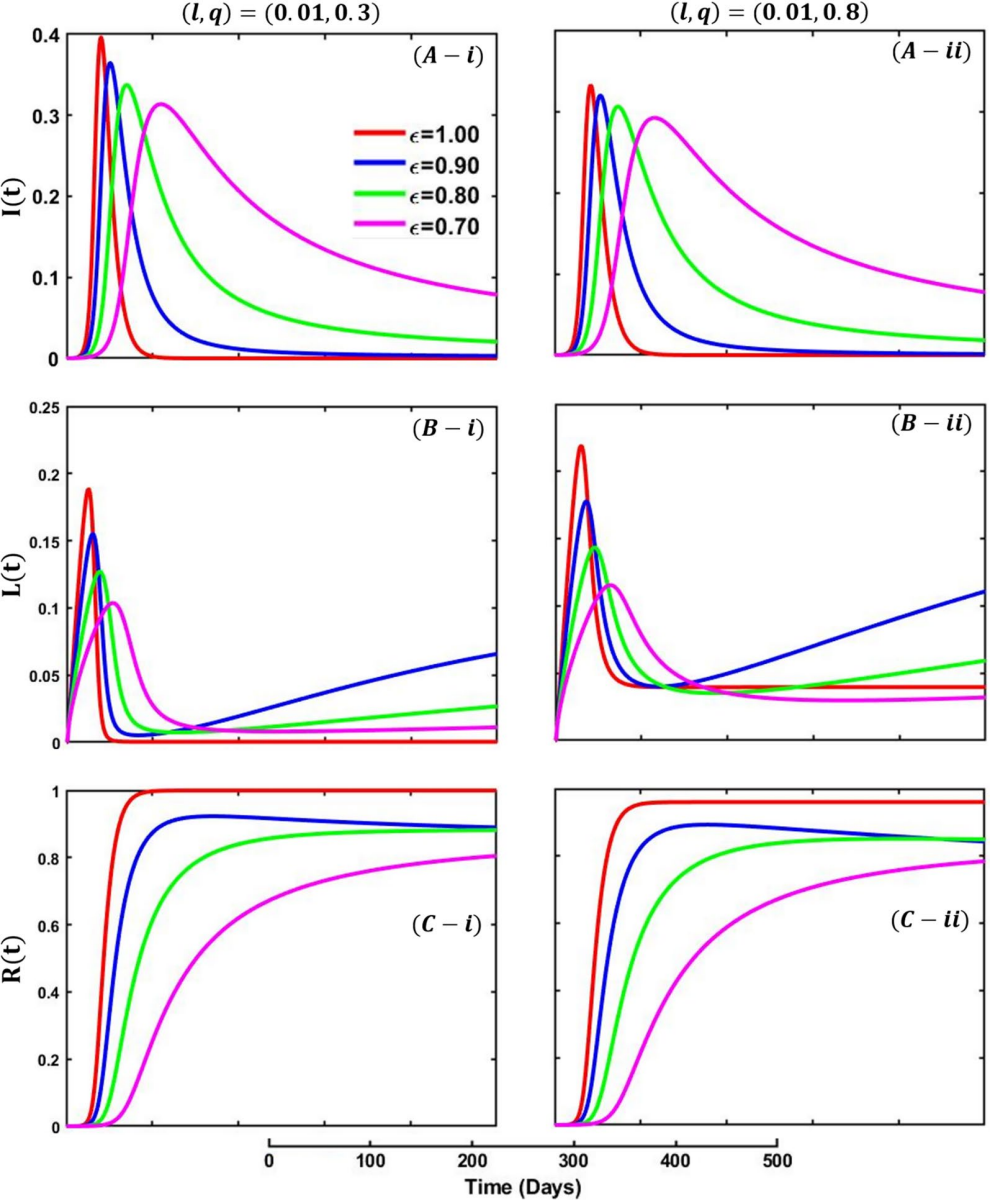
Presented is the effect of changing the fractional-order $\varepsilon =0.7, 08, 0.9, 1.0$ on the infected (Panel A-*), recovered (Panel B-*), and lockdown (Panel C-*) individuals. Subpanels (*-i), panel (*-ii), and panel (*-iii) exhibit the outcomes for lockdown level and lockdown maintenance factor $\left(l,q\right)=(\mathrm{0.01,0.3})$ and $(\mathrm{0.01,0.8})$, whereas the remaining parameters used are $\beta =1.0, \gamma =0.1, \alpha =\frac{1}{5},\eta =0.0, \delta =0.0,l=0.0$

Figure 10 depicts the fractional-order derivative simulation curve of infected (Panel A-*), vaccinated (Panel B-*), and recovered (Panel C-*) individuals for fractional-order $\varepsilon =0.7, 0.8, 0.9, 1.0$, whereas Fig. 11 represents infected (Panel A-*), lockdown (Panel B-*), and recovered (Panel C-*). In Fig. 10, panels (*-i), panel (*-ii), and panel (*-iii) show the results under the vaccine efficacy rate and vaccination rate as, $\left(\eta ,\delta \right)=\left(\mathrm{0.8,0.05}\right), (\mathrm{0.95,0.05})$ and $(\mathrm{0.95,0.1})$, respectively, with the remaining parameter settings $\beta =1.0, \gamma =0.1, \alpha =1/5,\eta =0.8, q=0.0,$ $l=0.0.$ On the other hand, in Fig. 11, panels (*-i) and (*-ii) exhibit the outcomes for lockdown level and lockdown maintenance factor $\left(l,q\right)=(\mathrm{0.01,0.3})$ and $(\mathrm{0.01,0.8})$,whereas the remaining parameters used are $\beta =1.0, \gamma =0.1, \alpha =1/5,\eta =0.0, \delta =0.0.$

Fractional-order models consider the possibility that individuals can be infected to a fractional degree rather than classified as either infected or not infected. It allows for a more detailed understanding of the epidemic, including the ability to capture subpopulations that may be more or less susceptible to the disease. Figure 10, panel A, shows that decreasing the fractional-order level delays the infection’s peak and makes the curves flatter. However, the vaccinated individuals illustrate the opposite trend; increasing the level of fractional-order decreases the vaccinated individuals as expected (panel C). On top of that, higher vaccine efficacy with higher vaccine intake decreases infection and recovers individuals as most people participated in the vaccine program. Finally, Fig. 11 presents the effect of fractional order on lockdown, whereas increasing values increase the peak of lockdown maintained people.

## Conclusion

Overcoming the hazardous situation of epidemic disease, we proposed $SLEIRV{E}_{V}{I}_{V}{R}_{V}$ epidemic model with a vaccination shortage. On top of that, the proposed model is biologically significant, and its solution is positive for all $t\ge 0$*.* Also, it is specified by the entire population $N\left(t\right)$ [12]. Moreover, discuss the point of equilibrium, ${R}_{0}, {R}_{e}$, the relationship between critical vaccine proportions, and the first and second derivatives of LF. Finally, we showed that the proposed model is globally stable. For numerical simulation, the finite difference method has been applied. Furthermore, the ABC fractional-order scheme has been used for fractional-order numerical simulation, which delayed the epidemic pick for lower-order values. In addition, while describing the real-world scenarios, the heat maps are pictured even though all are assumptions. Realistically, such analysis is essential for the current situation to improve the world’s real-life situation. Thus, our parametric study suggests that the combined effect of the non-pharmaceutical intervention, namely, lockdown and mass effective vaccination program, is much more effective for eradicating the disease from society and economic loss. The above results demonstrate that the lockdown level and more effective vaccination program with no shortage assist policymakers in discussing the best strategy for combating epidemic diseases.

Our study thoroughly analyses the impact of epidemic control strategies using three different frameworks: deterministic, heterogeneous network, and fractional order. By considering the same epidemic model, we sought to understand how vaccination and lockdown strategies interact to combat the spread of infectious diseases. The results of our research reveal intriguing insights that hold significant implications for policymakers and researchers. Through the deterministic approach, we comprehensively understood how conventional vaccination and lockdown measures can effectively curb the epidemic’s progression. Furthermore, exploring the fractional-order framework sheds light on the significance of considering vaccine availability and efficacy more nuancedly. The simulations demonstrated that a lower fractional-order could delay the epidemic’s peak, emphasizing the importance of strategically deploying vaccination resources. Additionally, incorporating a heterogeneous network offered valuable insights into the behavior of individuals in society during an epidemic. Understanding the dynamics of infection and control strategies through the average degree distribution highlighted the significance of community structure in shaping the outcomes of epidemic control measures. The findings from our study offer valuable guidance to policymakers and researchers, enabling them to make informed decisions and implement dynamic and effective measures to combat future epidemics. By recognizing the interplay between vaccination, lockdown, and the societal context, we can better tailor strategies to mitigate the impact of infectious diseases on a global scale. These insights can contribute significantly to public health efforts, safeguarding communities and saving lives. As we continue to face emerging infectious challenges, our research lays the foundation for evidence-based policies that prioritize both individual well-being and the collective health of our societies.

## Figures and Tables

**Fig. 12:**
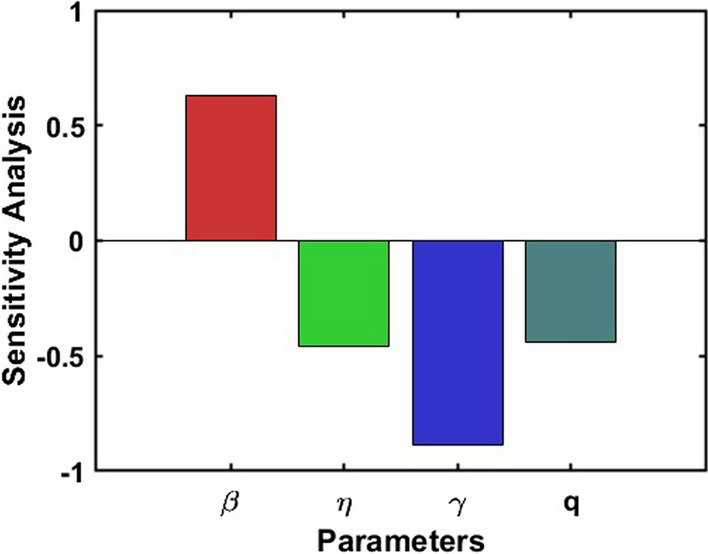
The reproduction number's partial rank correlation coefficients (PRCCs) and sensitivity to various parameters were assessed using baseline values from Table 2. The most influential parameters, indicated by their respective PRCC indices, are as follows: $\beta (0.638029)$, $\eta (-0.451558)$, $\gamma (-0.881569)$, and $q (-0.440801)$

## Data Availability

No data are used for this research work; all materials are included in manuscripts.

## Appendix

### Mathematical analysis

The present section studies the proposed model’s positivity and boundedness, basic $${(R}_{0})$$ reproduction number, the threshold of points of equilibrium (disease-free and endemic), stability of disease-free $${(E}_{0})$$ and endemic ($${E}_{*})$$ equilibrium point, effective $${(R}_{e})$$ reproduction number, the relation between $${R}_{0}$$ and crucial harmonies of vaccination, the first- and second-order Lyapunov functions (LF), existence, and uniqueness theorem for validating the model’s stability.

### Models positivity and boundedness [63–65]

Let the assumed state vector be represented as ${\varvec{x}} = \left( {{\varvec{x}}_{1} , {\varvec{x}}_{2} , {\varvec{x}}_{3} , {\varvec{x}}_{4} , {\varvec{x}}_{5} , {\varvec{x}}_{6} , {\varvec{x}}_{7} , {\varvec{x}}_{8} , {\varvec{x}}_{9} } \right) = \left( {S, L, E, I, R, V, E_{V} , I_{V} , R_{V} } \right)$. Then, Eq. (1) can be signed as some initial set of values.

12.1 $$\user2{x^{\prime}} = f\left( {{\varvec{x}}, t} \right), {\varvec{x}}\left( 0 \right) = {\varvec{x}}_{0} .$$

It is evident that the above-defined function f is locally Lipschitz for the first argument and continuous for the second in ${\mathbb{R}}^{9} \times {\mathbb{R}}$, which implies that the solution ${\varvec{x}}\left( t \right)$ holds in $t \in \left( {0, T} \right)$ for some $T,$ according to the system of nonlinear ODEs existence and uniqueness theorem [63, 66]. For the invariance of sets under a flow, we used the Bony–Brezis theorem [67]. The theorem applies to smooth manifolds. There are some corner points at the boundary of the invariant set. Shortly, by making an argument that if we have an initial condition at the corner points, it cannot go outside; for instance, the direction of the flow is towards the interior at those points. Thus, $T$ might be re-defined as the supremum overall mentioned periods. Finally, for $T < \infty$, $\mathop {\lim }\limits_{{t \to T^{ - } }} \parallel {\varvec{x}}\left( t \right)\parallel \to \infty .$

We have to show that under the flow ${\varvec{x}}\left( t \right)$ for $t \in \left( {0, T} \right),$ if

$${\varvec{x}}_{0} \in {\Lambda } = \left\{ {{\varvec{x}} \in {\mathbb{R}}^{9} :{\varvec{x}}_{i} \ge 0\;{\text{for}}\;i = 1,2,3, \cdots ,9, \mathop \sum \limits_{i = 1}^{9} {\varvec{x}}_{i} \le N\left( 0 \right)} \right\},$$

the set ${\Lambda }$ is positively invariant, which concludes that $T = \infty$, i.e., for initial conditions ${\varvec{x}}_{0} \in {\Lambda }$, the solution ${\varvec{x}}\left( t \right) \in {\Lambda }$ holds globally with time [63, 65, 66].

#### Theorem 1

The closed set.

$${\Lambda }: = \left\{ {{\varvec{x}} = \left( {S, L, E, I, R, V, E_{V} , I_{V} , R_{V} } \right) \in {\mathbb{R}}^{9} :{\varvec{x}}_{i} \ge 0\;{\text{for}}\;i = 1,2,3, \ldots ,9, \mathop \sum \limits_{i = 1}^{9} {\varvec{x}}_{i} \le N\left( 0 \right)} \right\},$$

is entirely uniform under the flow generated by Eqs. 1.1–1.9. As a result, solution ${\varvec{x}}\left( {\varvec{t}} \right) \in {\Lambda }$ occurs globally in the time given initial conditions ${\varvec{x}}_{0} \in {\Lambda }$.

#### Proof

Let the boundary segment is ${\Pi }_{i} , i = 1,2, \cdots , 10$.

$$\begin{aligned} {\Pi }_{i} & = \left\{ {{\varvec{x}} \in {\Lambda }: {\varvec{x}}_{{\varvec{i}}} = 0} \right\}, i = 1,2, \ldots ,9 \\ {\Pi }_{10} & = \left\{ {{\varvec{x}} \in {\Lambda }: \mathop \sum \limits_{i = 1}^{10} {\varvec{x}}_{i} = 0} \right\}. \\ \end{aligned}$$

It is evident that ${ }\partial {\Lambda } = \cup_{i = 1}^{10} {\Pi }_{i} .$

To complete the proof of the above invariance of the set ${\Lambda }$, it is enough to prove $\forall$ inward normal, ${\varvec{n}}.\user2{x^{\prime}}\left( t \right) \ge 0$ on $\partial {\Lambda }.$ The inward normal on ${\Pi }_{i} { }$ for $i = 1,2, \cdots , 9$ is unambiguously provided by ${\varvec{n}}_{i} = {\varvec{v}}_{i} = [0$, $\cdots$, 1, $\cdots$, 0], where only the $i$-component is nonzero, but an inward normal on ${\Pi }_{10}$ is given by ${\varvec{n}}_{10} = \left( { - 1,{ } - 1,{ } \cdots ,{ } - 1} \right)$.

Now, on ${\Pi }_{i} { }$ for $i = 1,2, \cdots ,9$

$$\begin{gathered} {\varvec{v}}_{1} .\user2{x^{\prime}} = l_{d} {\varvec{x}}_{2} \ge 0, \forall {\varvec{x}} \in {\Pi }_{1} , \hfill \\ {\varvec{v}}_{2} .\user2{x^{\prime}} = l{\varvec{x}}_{1} \ge 0, \forall {\varvec{x}} \in {\Pi }_{2} , \hfill \\ {\varvec{v}}_{3} .\user2{x^{\prime}} = \beta {\varvec{x}}_{1} \left( {{\varvec{x}}_{4} + {\varvec{x}}_{8} } \right) + \left( {1 - q} \right)\beta {\varvec{x}}_{2} \left( {{\varvec{x}}_{4} + {\varvec{x}}_{8} } \right) \ge 0, \forall {\varvec{x}} \in {\Pi }_{3} , \hfill \\ {\varvec{v}}_{4} .\user2{x^{\prime}} = \alpha {\varvec{x}}_{3} \ge 0,\forall {\varvec{x}} \in {\Pi }_{4} , \hfill \\ {\varvec{v}}_{5} .\user2{x^{\prime}} = \gamma {\varvec{x}}_{4} \ge 0, \forall {\varvec{x}} \in {\Pi }_{5} , \hfill \\ {\varvec{v}}_{6} .\user2{x^{\prime}} = \delta {\varvec{x}}_{1} \ge 0,\forall {\varvec{x}} \in {\Pi }_{6} , \hfill \\ {\varvec{v}}_{7} .\user2{x^{\prime}} = \left( {1 - \eta } \right)\beta {\varvec{x}}_{6} \left( {{\varvec{x}}_{4} + {\varvec{x}}_{8} } \right) \ge 0,\forall {\varvec{x}} \in {\Pi }_{7} , \hfill \\ {\varvec{v}}_{8} .\user2{x^{\prime}} = \alpha {\varvec{x}}_{7} \ge 0,\forall {\varvec{x}} \in {\Pi }_{8} , \hfill \\ {\varvec{v}}_{9} .\user2{x^{\prime}} = \gamma {\varvec{x}}_{8} \ge 0,\forall {\varvec{x}} \in {\Pi }_{9} , \hfill \\ \end{gathered}$$

while ${\text{N}} = \mathop \sum \limits_{i = 1}^{9} {\mathbf{x}}_{i}$ is readily seen to satisfy $\frac{dN}{{dt}} = 0$, which follows that on ${\Pi }_{10}$ such that

$${\varvec{n}}_{10} .\user2{x^{\prime}} = 0.$$

Thus, for given initial conditions ${\varvec{x}}_{0} \in {\Lambda }$, solution ${\varvec{x}}\left( t \right) \in {\Lambda }$ holds globally over time on the positively invariant domain $\Lambda .$

### Derivation of the basic reproduction number ${(R}_{0})$

Before starting the formal analysis, we will describe how the so-called basic reproduction number ${(R}_{0})$ is computed in detail, which is vital in epidemiological modeling because it has been found to help comprehend stability conditions. It has been demonstrated that stability conditions can be expected if this number is less than one, but instability conditions occur if it is more significant than one. Nevertheless, it has been emphasized that the value can be derived using various approaches; among them, the next-generation matrix techniques [68] are well-established. Thus, the detailed process $FV^{ - 1} \left( { \equiv F_{1} V_{1}^{ - 1} ,F_{2} V_{2}^{ - 1} } \right)$ of finding the value of the basic reproduction number $(R_{0} )$ is

$$F_{1} = \left[ {\begin{array}{*{20}c} 0 & {\beta + \left( {1 - q} \right)\beta } \\ 0 & 0 \\ \end{array} } \right], V_{1} = \left[ {\begin{array}{*{20}c} \alpha & 0 \\ { - \alpha } & \gamma \\ \end{array} } \right].$$

$$\therefore F_{1} V_{1}^{ - 1} = \frac{1}{\alpha \gamma }\left[ {\begin{array}{*{20}c} {\alpha \beta } & {\alpha \left\{ {\beta + \left( {1 - q} \right)\beta } \right\}} \\ 0 & 0 \\ \end{array} } \right].$$

The eigenvalues of $F_{1} V_{1}^{ - 1}$ are $\lambda_{1} = \frac{\beta }{\gamma } and \lambda_{2} = \frac{{\beta + \left( {1 - q} \right)\beta }}{\gamma }.$

Correspondingly, for the vaccination part,

$$F_{2} = \left[ {\begin{array}{*{20}c} 0 & {\beta \left( {1 - \eta } \right)} \\ 0 & 0 \\ \end{array} } \right], V_{2} = \left[ {\begin{array}{*{20}c} \alpha & 0 \\ { - \alpha } & \gamma \\ \end{array} } \right].$$

$$\therefore F_{2} V_{2}^{ - 1} = \frac{1}{\alpha \gamma }\left[ {\begin{array}{*{20}c} {\alpha \beta } & {\alpha \left( {1 - \eta } \right)\beta } \\ 0 & 0 \\ \end{array} } \right].$$

The eigenvalues of $F_{2} V_{2}^{ - 1}$ are $\lambda_{3} = \frac{\beta }{\gamma }\;{\text{and}}\;\lambda_{4} = \frac{{\left( {1 - \eta } \right)\beta }}{\gamma } .$

Thus, the basic reproduction number $(R_{0} )$ is

12.2 $$R_{0} = \frac{\beta }{\gamma } + \frac{{\left( {1 - q} \right)\beta }}{\gamma } + \frac{{\left( {1 - \eta } \right)\beta }}{\gamma }.$$

### Analysis of disease-free and endemic equilibrium point threshold

For this model, the disease-free equilibrium point threshold is the solution of the nonlinear system of Eq. (1). Thus,

$$\frac{{{\text{d}}S}}{{{\text{d}}t}} = \frac{{{\text{d}}L}}{{{\text{d}}t}} = \frac{{{\text{d}}E}}{{{\text{d}}t}} = \frac{{{\text{d}}I}}{{{\text{d}}t}} = \frac{{{\text{d}}R}}{{{\text{d}}t}} = \frac{{{\text{d}}V}}{{{\text{d}}t}} = \frac{{E_{V} }}{{{\text{d}}t}} = \frac{{I_{V} }}{{{\text{d}}t}} = \frac{{R_{V} }}{{{\text{d}}t}} = 0,$$

gives $E_{0} = \left( {{\text{S}}^{0} ,{ }0,{ }0,{ }0,{ }0,{\text{ V}}^{0} ,{ }0,{ }0,{ }0} \right),{ }$ the disease-free equilibrium point threshold of the current model.

The proposed model endemic equilibrium point $E_{*} = \left( {S^{*} ,L^{*} ,E^{*} ,I^{*} ,R^{*} ,V^{*} ,E_{{v^{*} }} ,I_{{v^{*} }} ,R_{{v^{*} }} } \right)$ is a solution of the following system:

$$\begin{aligned} 0 & = - \beta S\left( t \right)\left( {I\left( t \right) + I_{V} \left( t \right)} \right) - \delta S\left( t \right) - lS\left( t \right) + l_{d} L\left( t \right), \\ 0 & = lS\left( t \right) - \left( {1 - q} \right)\beta L\left( t \right)\left( {I\left( t \right) + I_{V} \left( t \right)} \right) - l_{d} L\left( t \right), \\ 0 & = \beta S\left( t \right)\left( {I\left( t \right) + I_{V} \left( t \right)} \right) + \left( {1 - q} \right)\beta L\left( t \right)\left( {I\left( t \right) + I_{V} \left( t \right)} \right) - \alpha E\left( t \right), \\ 0 & = \alpha E\left( t \right) - \gamma I\left( t \right), \\ 0 & = \gamma I\left( t \right), \\ 0 & = \delta S\left( t \right) - \left( {1 - \eta } \right)\beta V\left( t \right)\left( {I\left( t \right) + I_{V} \left( t \right)} \right), \\ 0 & = \left( {1 - \eta } \right)\beta V\left( t \right)\left( {I\left( t \right) + I_{V} \left( t \right)} \right) - \alpha E_{V} \left( t \right), \\ 0 & = \alpha E_{V} \left( t \right) - \gamma I_{V} \left( t \right), \\ 0 & = \gamma I_{V} \left( t \right). \\ \end{aligned}$$

Solving the above system of equations setting $I \ne 0,I_{V} \ne 0$, the endemic equilibrium point is.

$$E_{*} = \left( {S^{*} ,L^{*} ,E^{*} ,I^{*} ,R^{*} ,V^{*} ,E_{{v^{*} }} ,I_{{v^{*} }} ,R_{{v^{*} }} } \right),$$

where

$$S^{*} = \frac{{\gamma I^{*} }}{{\beta I^{*} + \delta + l}},L^{*} = \frac{\gamma }{{\left( {1 - q} \right)\beta }} - \frac{{\gamma I^{*} }}{{\left( {1 - q} \right)\left( {\beta I^{*} + \delta + l} \right)}},E^{*} = \frac{{l_{d} L^{*} }}{\alpha },I^{*} = \frac{{l_{d} L^{*} }}{\gamma },R^{*} = l_{d} L^{*}$$

$$V^{*} = \frac{\gamma }{{\left( {1 - \eta } \right)\beta }},E_{{v^{*} }} = \frac{{\delta S^{*} }}{\alpha },I_{{v^{*} }} = \frac{{\delta S^{*} }}{\gamma },R_{{v^{*} }} = \delta S^{*} .$$

### Stability of disease-free $(E_{0} )$ equilibrium point

In this section, we will show that for ${R}_{0}<1$ and ${R}_{0}>1$ the disease-free ${(E}_{0})$ equilibrium point is asymptotically stable locally. The disease will be eradicated and continue biologically in society when the primary reproduction number is less than and more significant than unity.

#### Theorem 2

If ${R}_{0}<1$, then the unique disease-free equilibrium ${E}_{0}$ is locally asymptotically stable. If ${R}_{0}>1$, the unique disease-free equilibrium is unstable.

#### Proof

To validate the local stability, the Jacobian matrix of the proposed system (1) is.

$$J = \left[ {\begin{array}{*{20}c} { - \beta (I + I_{V} ) - \delta - l} & {l_{d} } & 0 & { - \beta S} & 0 & 0 & 0 & { - \beta S} & 0 \\ l & { - \beta (1 - q)(I + I_{V} ) - l_{d} } & 0 & { - \beta (1 - q)L} & 0 & 0 & 0 & { - \beta (1 - q)L} & 0 \\ {\beta (I + I_{V} )} & {\beta (1 - q)(I + I_{V} )} & { - \alpha } & {\beta S + \beta (1 - q)L} & 0 & 0 & 0 & {\beta S + \beta (1 - q)L} & 0 \\ 0 & 0 & \alpha & { - \gamma } & 0 & 0 & 0 & 0 & 0 \\ 0 & 0 & 0 & \gamma & 0 & 0 & 0 & 0 & 0 \\ \delta & 0 & 0 & { - \beta (1 - \eta )V} & 0 & { - \beta (1 - \eta )(I + I_{V} )} & 0 & { - \beta (1 - \eta )V} & 0 \\ 0 & 0 & 0 & {\beta (1 - \eta )V} & 0 & {\beta (1 - \eta )(I + I_{V} )} & { - \alpha } & {\beta (1 - \eta )V} & 0 \\ 0 & 0 & 0 & 0 & 0 & 0 & \alpha & { - \gamma } & 0 \\ 0 & 0 & 0 & 0 & 0 & 0 & 0 & \gamma & 0 \\ \end{array} } \right].$$

At the DFE point ${E}_{0},$ we have

$$J(E_{0} ) = \left[ {\begin{array}{*{20}c} { - \delta - l} & {\,\,\,\,l_{d} } & {\,\,\,0} & {\,\,\,0} & 0 & 0 & 0 & 0 & 0 \\ l & { - l_{d} } & {\,\,\,0} & {\,\,\,0} & 0 & 0 & 0 & 0 & 0 \\ 0 & 0 & { - \alpha } & {\,\,\,0} & 0 & 0 & 0 & 0 & 0 \\ 0 & 0 & {\,\,\,\alpha } & { - \gamma } & 0 & 0 & 0 & 0 & 0 \\ 0 & 0 & {\,\,\,0} & {\,\,\,\gamma } & 0 & 0 & 0 & 0 & 0 \\ \delta & 0 & {\,\,\,0} & {\,\,\,0} & 0 & 0 & 0 & 0 & 0 \\ 0 & 0 & {\,\,\,0} & {\,\,\,0} & 0 & 0 & { - \alpha } & 0 & 0 \\ 0 & 0 & {\,\,\,0} & {\,\,\,0} & 0 & 0 & {\,\,\,\alpha } & { - \gamma } & 0 \\ 0 & 0 & {\,\,0} & {\,\,\,0} & 0 & 0 & {\,\,\,0} & {\,\,\,\gamma } & 0 \\ \end{array} } \right].$$

The characteristic equation $\left| {J\left( {E_{0} } \right) - \lambda I} \right| = 0$ has nine roots, which are $\lambda_{1} = \lambda_{2} = \lambda_{3} = 0, \lambda_{4} = \lambda_{5} = - \alpha ,$ $\lambda_{6} = \lambda_{7} = - \gamma ,$ i.e., the first seven eigenvalues are less or equal to zero. Thus, the stability of the DFE point depends on the remaining two roots ($\lambda_{8} , \lambda_{9} ),$ the solution of the following quadratic characteristic equation:

$$\lambda^{2} + a_{1} \lambda + a_{2} = 0,$$

where

$$\begin{aligned} a_{1} & = \delta + l + l_{d} , \\ a_{2} & = \delta l_{d} . \\ \end{aligned}$$

It is clear that the coefficients represented above are positive, i.e., $a_{1} > 0$ and $a_{2} > 0$. As a result, it implies that $a_{i} > 0$ for $i = 1,2$ are positive and follow the Routh–Hurwitz criterion [7]. On top of that, we conclude that all eigenvalues $\lambda_{k} \left( {k = 4,5, \ldots ,9} \right)$ calculated from the Jacobian matrix at the DFE point have negative real parts. Therefore, the model is locally asymptotically stable at the unique DFE point whenever $R_{0} < 1$ and unstable whenever $R_{0} > 1$.

#### Theorem 3

The endemic equilibrium point $E^{*}$ is locally asymptotically stable and unstable whenever $R_{0} > 1.$

**Proof**

The endemic equilibrium point $E_{*} ,$ the desired Jacobian matrix is

$$J = \left[ {\begin{array}{*{20}c} { - \beta (I^{*} + I_{V}^{*} ) - \delta - l} & {l_{d} } & 0 & { - \beta S^{*} } & 0 & 0 & 0 & { - \beta S^{*} } & 0 \\ l & { - \beta (1 - q)(I^{*} + I_{V}^{*} ) - l_{d} } & 0 & { - \beta (1 - q)L^{*} } & 0 & 0 & 0 & { - \beta (1 - q)L^{*} } & 0 \\ {\beta (I^{*} + I_{V}^{*} )} & {\beta (1 - q)(I^{*} + I_{V}^{*} )} & { - \alpha } & {\beta S + \beta (1 - q)L^{*} } & 0 & 0 & 0 & {\beta S + \beta (1 - q)L^{*} } & 0 \\ 0 & 0 & \alpha & { - \gamma } & 0 & 0 & 0 & 0 & 0 \\ 0 & 0 & 0 & \gamma & 0 & 0 & 0 & 0 & 0 \\ \delta & 0 & 0 & { - \beta (1 - \eta )V^{*} } & 0 & { - \beta (1 - \eta )(I^{*} + I_{V}^{*} )} & 0 & { - \beta (1 - \eta )V^{*} } & 0 \\ 0 & 0 & 0 & {\beta (1 - \eta )V^{*} } & 0 & {\beta (1 - \eta )(I^{*} + I_{V}^{*} )} & { - \alpha } & {\beta (1 - \eta )V^{*} } & 0 \\ 0 & 0 & 0 & 0 & 0 & 0 & \alpha & { - \gamma } & 0 \\ 0 & 0 & 0 & 0 & 0 & 0 & 0 & \gamma & 0 \\ \end{array} } \right].$$

The roots of the characteristic equations $|J(E_{*} - \lambda I_{7} |$ satisfy the following equation:

12.3 $$\lambda^{5} + a_{4} \lambda^{4} + a_{3} \lambda^{3} + a_{2} \lambda^{2} + a_{1} \lambda + a_{0} = 0,$$

where,

$$a_{4} = - A_{11} + A_{13} - A_{16} + \delta + \omega ,$$

$$a_{3} = - A_{11} A_{13} + A_{13} A_{14} - A_{12} A_{15} + A_{11} A_{16} - A_{13} f - a\delta + A_{13} \delta - f\delta - a\omega + A_{13} \omega - A_{16} \omega + \delta \omega ,$$

$$\begin{aligned} a_{2} & = - A_{11} A_{13} A_{14} - A_{12} A_{13} A_{15} + A_{11} A_{13} A_{16} - A_{12} A_{13} x - A_{11} A_{13} \delta + A_{13} A_{14} \delta - A_{12} A_{15} \delta \\ & \quad + A_{11} A_{16} \delta - A_{13} A_{16} \delta - A_{11} A_{13} \omega + A_{13} A_{14} \omega - A_{12} A_{15} \omega + A_{11} A_{16} \omega \\ & \quad - A_{13} A_{16} \omega - A_{15} \gamma \omega - A_{11} \delta \omega + A_{13} \delta \omega - A_{16} \delta \omega , \\ \end{aligned}$$

$$\begin{aligned} a_{1} & = - A_{11} A_{13} A_{14} \delta - A_{12} A_{13} A_{15} \delta + A_{11} A_{13} A_{16} \delta - A_{12} A_{13} x\delta - A_{12} A_{13} e\omega + A_{11} A_{13} A_{16} \omega \\ & \quad - A_{12} A_{13} x\omega - A_{13} A_{15} \gamma \omega - A_{13} x\gamma \omega - A_{11} A_{13} \delta \omega + A_{13} A_{14} \delta \omega - A_{12} A_{15} \delta \omega \\ & \quad + A_{11} A_{16} \delta \omega - A_{13} A_{16} \delta \omega - A_{15} \gamma \delta \omega - A_{15} \delta \tau \omega - A_{11} A_{13} A_{14} \omega , \\ \end{aligned}$$

$$a_{0} = - A_{13} \left( {A_{11} A_{14} \delta \omega + A_{12} A_{15} \delta \omega - A_{11} A_{16} \delta \omega + A_{12} x\delta \omega + A_{15} \gamma \delta \omega + x\gamma \delta \omega + A_{15} \delta \tau \omega + x\delta \tau \omega } \right),$$

$$A_{11} = - \beta I^{*} - x,$$

$$A_{12} = - \beta S^{*} ,$$

$$A_{13} = \left( {1 - \eta } \right)\beta I^{*} ,$$

$$A_{14} = \left( {1 - \eta } \right)\beta V^{*} ,$$

$$A_{15} = \beta I^{*} ,$$

$$A_{16} = \beta S^{*} + \left( {1 - \eta } \right)\beta V^{*} - \gamma - \tau .$$

It is simple to demonstrate that all of the roots of Eq. (12.3) will have a negative real portion if ${R}_{0}>1$ and that the coefficients of (12.3) will fulfill the Routh–Hurwitz condition [7]. The endemic equilibrium point will thus be locally asymptotically stable for ${R}_{0}>1$.

### Derivation of the effective reproduction number ${(R}_{e})$

In general, a population in the real world can infrequently be entirely susceptible to infection. Some contacts will be immune because of previous infections, which have provided long-term immunity or the consequence of prior vaccinations. Consequently, all communications will not infect, and the mean per secondary case of disease is less than the number of basic reproduction numbers. Thus, the effective reproductive number (${R}_{e}$) is the mean per infectious case in a population of susceptible and non-susceptible people. When ${R}_{e}>1,$ the number of cases will increase and start an epidemic. When ${R}_{e}=1$, the disease is endemic, and when ${R}_{e}<1$, there will be a decline in the number of cases. The effective reproduction number is sensitive to the multiple of the basic reproduction number and the fraction of the host population. Therefore,

12.4 $$R_{e} = \frac{\beta }{\gamma }S\left( t \right) + \frac{{\left( {1 - q} \right)\beta }}{\gamma }L\left( t \right) + \frac{{\left( {1 - \eta } \right)\beta }}{\gamma }V\left( t \right).$$

### Relation between $R_{0}$ and crucial harmonies of vaccination

In the recommended model, $\frac{{\beta S\left( t \right)\left( {I\left( t \right) + I_{V} \left( t \right)} \right) }}{N}$ is used to compute the sum of secondary infections of susceptible and vaccinated infected people per unit of time. On top of that, it is equals $\frac{\beta S\left( t \right)}{N}$ for the single infected individual. Furthermore, $\frac{1}{\gamma }$ and $\frac{\beta }{\gamma }$ indicates the lifespan of an infectious individual and the total secondary infections of susceptible individuals that may produce one infected individual in a disease-free society, respectively, whereas for vaccinated individuals, both of the values are $\frac{1}{\gamma }$ and $\frac{{\left( {1 - \eta } \right)\beta }}{\gamma }$. Analogously, secondary infections of vaccinated individuals equal $\frac{\beta S(t){I}_{V}(t)}{N}$ and per unit of time, the sum is $\frac{\beta S(t)}{N}$. Realistically, due to the mass vaccination rate $\frac{1}{\delta },$ the basic reproduction number must be a decreasing function. Opposite scenarios for less vaccine efficacy rate$\left(1-\eta \right)$.

### The first derivative of the Lyapunov function (*LF*)

Let us assume models independent variables for the endemic LF, $\left\{S, L, E,I,R, V,{E}_{V},{I}_{V},{R}_{V}\right\}, {L}_{f}<0$ is the detrimental equilibrium point ${E}_{*}$.

#### Theorem 4

For the value of the basic reproductive number ${R}_{0}-1>0$, the proposed $SLEIRV{E}_{V}{I}_{V}{R}_{V}$ models endemic equilibrium point ${E}_{*}$ is globally asymptotically stable.

#### Proof

The LF may be represented as follows to prove the theorem above:

12.5 $$\begin{aligned} & L_{f} \left( {S, L, E,I,R, V,E_{V} ,I_{V} ,R_{V} } \right) = \left( {S - S^{*} - S^{*} \log \frac{{S^{*} }}{S}} \right) + \left( {L - L^{*} - L^{*} \log \frac{{L^{*} }}{L}} \right) \\ & \quad + \left( {E - E^{*} - E^{*} \log \frac{{E^{*} }}{E}} \right) + \left( {I - I^{*} - I^{*} \log \frac{{I^{*} }}{I}} \right) + \left( {R - R^{*} - R^{*} \log \frac{{R^{*} }}{R}} \right) \\ & \left( {V - V^{*} - V\log \frac{{V^{*} }}{V}} \right) + \left( {E_{V} - E_{V}^{*} - E_{V}^{*} \log \frac{{E_{V}^{*} }}{{E_{V} }}} \right) + \left( {I_{V} - I_{V}^{*} - I_{V}^{*} \log \frac{{I_{V}^{*} }}{{I_{V} }}} \right) \\ & \quad + \left( {R_{V} - R_{V}^{*} - R_{V}^{*} \log \frac{{R_{V}^{*} }}{{R_{V} }}} \right). \\ \end{aligned}$$

Differentiating both sides in terms of *t*, we get,

12.6 $$\begin{aligned} \frac{{{\text{d}}L_{f} }}{{{\text{d}}t}} & = \dot{L}_{f} = \left( {\frac{{S - S^{*} }}{S}} \right)\dot{S} + \left( {\frac{{L - L^{*} }}{L}} \right)\dot{L} + \left( {\frac{{E - E^{*} }}{E}} \right)\dot{E} + \left( {\frac{{I - I^{*} }}{I}} \right)\dot{I} + \left( {\frac{{R - R^{*} }}{R}} \right)\dot{R} \\ & \quad + \left( {\frac{{V - V^{*} }}{V}} \right)\dot{V} + \left( {\frac{{E_{V} - E_{V}^{*} }}{{E_{V} }}} \right)\dot{E}_{V} + \left( {\frac{{I_{V} - I_{V}^{*} }}{{I_{V} }}} \right)\dot{I}_{V} + \left( {\frac{{R_{V} - R_{V}^{*} }}{{R_{V} }}} \right)\dot{R}_{V} . \\ \end{aligned}$$

From Eq. (1), putting the value of $\dot{S}, \dot{L}, \dot{E},\dot{I},\dot{V},\dot{E}_{V} ,\dot{I}_{V} ,\dot{R}_{V}$ in Eq. (12.6), we have,

12.7 $$\begin{aligned} \frac{{{\text{d}}L_{f} }}{{{\text{d}}t}} & = \left( {\frac{{S - S^{*} }}{S}} \right)\left\{ { - \beta S\left( {I + I_{V} } \right) - \delta S - lS + l_{d} L} \right\} \\ & \quad + \left( {\frac{{L - L^{*} }}{L}} \right)\left\{ {lS - \left( {1 - q} \right)\beta L\left( {I + I_{V} } \right) - l_{d} L} \right\} \\ & \quad + \left( {\frac{{E - E^{*} }}{E}} \right)\left\{ {\beta S\left( {I + I_{V} } \right) + \left( {1 - q} \right)\beta L\left( {I + I_{V} } \right) - \alpha E} \right\} \\ & \quad + \left( {\frac{{I - I^{*} }}{I}} \right)\left( {\alpha E - \gamma I} \right) + \left( {\frac{{R - R^{*} }}{R}} \right)\gamma I \\ & \quad + \left( {\frac{{V - V^{*} }}{V}} \right)\left\{ {\delta S - \left( {1 - \eta } \right)\beta V\left( {I + I_{V} } \right)} \right\} \\ & \quad + \left( {\frac{{E_{V} - E_{V}^{*} }}{{E_{V} }}} \right)\left\{ {\left( {1 - \eta } \right)\beta V\left( {I + I_{V} } \right) - \alpha E_{V} } \right\} \\ & \quad + \left( {\frac{{I_{V} - I_{V}^{*} }}{{I_{V} }}} \right)\left( {\alpha E_{V} - \gamma I_{V} } \right) \\ & \quad + \left( {\frac{{R_{V} - R_{V}^{*} }}{{R_{V} }}} \right)\gamma I_{V} . \\ \end{aligned}$$

In Eq. (12.7), substitute $S - S^{*} ,L - L^{*} ,E - E^{*} ,I - I^{*} ,V - V^{*} ,E_{V} - E_{V}^{*} ,I_{V} - I_{V}^{*} ,R_{V} - R_{V}^{*}$ instead of $S, L, E,I,R, V,E_{V} ,I_{V} ,R_{V} .$ Then,

12.8 $$\begin{aligned} \frac{{{\text{d}}L_{f} }}{{{\text{d}}t}} & = \left( {\frac{{S - S^{*} }}{S}} \right)\left[ { - \beta \left( {S - S^{*} } \right)\left\{ {\left( {I - I^{*} } \right) + \left( {I_{V} - I_{V}^{*} } \right)} \right\} - \delta \left( {S - S^{*} } \right) - l\left( {S - S^{*} } \right) + l_{d} \left( {L - L^{*} } \right)} \right] \\ & \quad + \left( {\frac{{L - L^{*} }}{L}} \right)\left[ {l\left( {S - S^{*} } \right) - \left( {1 - q} \right)\beta \left( {L - L^{*} } \right)\left\{ {\left( {I - I^{*} } \right) + \left( {I_{V} - I_{V}^{*} } \right)} \right\} - l_{d} \left( {L - L^{*} } \right)} \right] \\ & \quad + \left( {\frac{{E - E^{*} }}{E}} \right)[\beta \left( {S - S^{*} } \right)\left\{ {\left( {I - I^{*} } \right) + \left( {I_{V} - I_{V}^{*} } \right)} \right\} + \left( {1 - q} \right)\beta \left( {L - L^{*} } \right)\left\{ {\left( {I - I^{*} } \right) + \left( {I_{V} - I_{V}^{*} } \right)} \right\} \\ & \quad - \alpha \left( {E - E^{*} } \right)] + \left( {\frac{{I - I^{*} }}{I}} \right)\left\{ {\alpha \left( {E - E^{*} } \right) - \gamma \left( {I - I^{*} } \right)} \right\} + \left( {\frac{{R - R^{*} }}{R}} \right)\gamma \left( {I - I^{*} } \right) \\ & \quad + \left( {\frac{{V - V^{*} }}{V}} \right)\left[ {\delta \left( {S - S^{*} } \right) - \left( {1 - \eta } \right)\beta \left( {V - V^{*} } \right)\left\{ {\left( {I - I^{*} } \right) + \left( {I_{V} - I_{V}^{*} } \right)} \right\}} \right] \\ & \quad + \left( {\frac{{E_{V} - E_{V}^{*} }}{{E_{V} }}} \right)\left[ {\left( {1 - \eta } \right)\beta \left( {V - V^{*} } \right)\left\{ {\left( {I - I^{*} } \right) + \left( {I_{V} - I_{V}^{*} } \right)} \right\} - \alpha \left( {E_{V} - E_{V}^{*} } \right)} \right] \\ & \quad + \left( {\frac{{I_{V} - I_{V}^{*} }}{{I_{V} }}} \right)\left\{ {\alpha \left( {E_{V} - E_{V}^{*} } \right) - \gamma \left( {I_{V} - I_{V}^{*} } \right)} \right\} \\ & \quad + \left( {\frac{{R_{V} - R_{V}^{*} }}{{R_{V} }}} \right)\gamma \left( {I_{V} - I_{V}^{*} } \right). \\ \end{aligned}$$

After simplifying, we may write,

12.9 $$\frac{{{\text{d}}L_{f} }}{{{\text{d}}t}} = {\Pi }_{1} - {\Pi }_{2} ,$$

where

$$\begin{aligned} {\Pi }_{1} & = \beta \frac{{\left( {S - S^{*} } \right)^{2} }}{S}I^{*} + \beta \frac{{\left( {S - S^{*} } \right)^{2} }}{S}I_{V}^{*} + l_{d} L + l_{d} \frac{{S^{*} }}{S}L^{*} + lS + l\frac{{L^{*} }}{L}S^{*} + \beta \left( {1 - q} \right)LI^{*} + \beta \left( {1 - q} \right)LI_{V}^{*} \\ & \quad + \beta \left( {1 - q} \right)L^{*} I + \beta \left( {1 - q} \right)L^{*} I_{V} + \beta \left( {1 - q} \right)\frac{{E^{*} }}{E}LI + \beta \left( {1 - q} \right)\frac{{E^{*} }}{E}LI_{V} + \beta \left( {1 - q} \right)\frac{{E^{*} }}{E}L^{*} I^{*} \\ & \quad + \beta \left( {1 - q} \right)\frac{{E^{*} }}{E}L^{*} I_{V}^{*} + \beta SI + \beta SI_{V} + \beta S^{*} I^{*} + \beta S^{*} I_{V}^{*} + \beta \frac{{E^{*} }}{E}SI^{*} + \beta \frac{{E^{*} }}{E}SI_{V}^{*} + \beta \frac{{E^{*} }}{E}S^{*} I + \beta \frac{{E^{*} }}{E}S^{*} I_{V} \\ & \quad + \beta \left( {1 - q} \right)LI + \beta \left( {1 - q} \right)LI_{V} + \beta \left( {1 - q} \right)L^{*} I^{*} + \beta \left( {1 - q} \right)L^{*} I_{V}^{*} + \beta \left( {1 - q} \right)\frac{{E^{*} }}{E}LI^{*} + \beta \left( {1 - q} \right)\frac{{E^{*} }}{E}LI_{V}^{*} \\ & \quad + \beta \left( {1 - q} \right)\frac{{E^{*} }}{E}L^{*} I + \beta \left( {1 - q} \right)\frac{{E^{*} }}{E}L^{*} I_{V} + \alpha E + \alpha \frac{{I^{*} }}{I}E^{*} + \gamma I + \gamma \frac{{R^{*} }}{R}I^{*} + \delta S + \delta \frac{{V^{*} }}{V}S^{*} \\ & \quad + \beta \left( {1 - \eta } \right)\frac{{\left( {V - V^{*} } \right)^{2} }}{V}I^{*} + \beta \left( {1 - \eta } \right)\frac{{\left( {V - V^{*} } \right)^{2} }}{V}I_{V}^{*} + \beta \left( {1 - \eta } \right)\frac{{E_{V}^{*} }}{{E_{V} }}VI^{*} + \beta \left( {1 - \eta } \right)\frac{{E_{V}^{*} }}{{E_{V} }}VI_{V}^{*} \\ & \quad + \beta \left( {1 - \eta } \right)\frac{{E_{V}^{*} }}{{E_{V} }}V^{*} I + \beta \left( {1 - \eta } \right)\frac{{E_{V}^{*} }}{{E_{V} }}V^{*} I_{V} + \gamma I_{V} + \gamma \frac{{R_{V}^{*} }}{{R_{V} }}I_{V}^{*} + \alpha E_{V} + \alpha \frac{{I_{V}^{*} }}{{I_{V} }}E_{V}^{*} , \\ \end{aligned}$$

and

$$\begin{aligned} {\Pi }_{2} & = \beta \frac{{\left( {S - S^{*} } \right)^{2} }}{S}I + \beta \frac{{\left( {S - S^{*} } \right)^{2} }}{S}I_{V} + \left( {\delta + l} \right)\frac{{\left( {S - S^{*} } \right)^{2} }}{S} + l_{d} L^{*} + l_{d} \frac{{S^{*} }}{S}L + lS^{*} + l\frac{{L^{*} }}{L}S \\ & \quad + \beta \left( {1 - q} \right)LI + \beta \left( {1 - q} \right)LI_{V} + \beta \left( {1 - q} \right)L^{*} I^{*} + \beta \left( {1 - q} \right)L^{*} I_{V}^{*} + \beta \left( {1 - q} \right)\frac{{E^{*} }}{E}LI^{*} \\ & \quad + \beta \left( {1 - q} \right)\frac{{E^{*} }}{E}LI_{V}^{*} + \beta \left( {1 - q} \right)\frac{{E^{*} }}{E}L^{*} I + \beta \left( {1 - q} \right)\frac{{E^{*} }}{E}L^{*} I_{V} + l_{d} \frac{{\left( {L - L^{*} } \right)^{2} }}{L} + \beta SI^{*} \\ & \quad + \beta SI_{V}^{*} + \beta S^{*} I + \beta S^{*} I_{V} + \beta \frac{{E^{*} }}{E}SI + \beta \frac{{E^{*} }}{E}SI_{V} + \beta \frac{{E^{*} }}{E}S^{*} I^{*} + \beta \frac{{E^{*} }}{E}S^{*} I_{V}^{*} + \beta \left( {1 - q} \right)LI^{*} \\ & \quad + \beta \left( {1 - q} \right)LI_{V}^{*} + \beta \left( {1 - q} \right)L^{*} I + \beta \left( {1 - q} \right)L^{*} I_{V} + \beta \left( {1 - q} \right)\frac{{E^{*} }}{E}LI + \beta \left( {1 - q} \right)\frac{{E^{*} }}{E}LI_{V} \\ & \quad + \beta \left( {1 - q} \right)\frac{{E^{*} }}{E}L^{*} I^{*} + \beta \left( {1 - q} \right)\frac{{E^{*} }}{E}L^{*} I_{V}^{*} + \alpha \frac{{\left( {E - E^{*} } \right)^{2} }}{E} + \alpha E^{*} + \alpha \frac{{I^{*} }}{I}E \\ & \quad + \gamma \frac{{\left( {I - I^{*} } \right)^{2} }}{I} + \gamma I^{*} + \gamma \frac{{R^{*} }}{R}I + \delta S^{*} + \delta \frac{{V^{*} }}{V}S + \beta \left( {1 - \eta } \right)\frac{{\left( {V - V^{*} } \right)^{2} }}{V}I + \beta \left( {1 - \eta } \right)\frac{{\left( {V - V^{*} } \right)^{2} }}{V}I_{V} \\ & \quad + \beta \left( {1 - \eta } \right)\frac{{E_{V}^{*} }}{{E_{V} }}VI + \beta \left( {1 - \eta } \right)\frac{{E_{V}^{*} }}{{E_{V} }}VI_{V} + \beta \left( {1 - \eta } \right)\frac{{E_{V}^{*} }}{{E_{V} }}V^{*} I^{*} \\ & \quad + \beta \left( {1 - \eta } \right)\frac{{E_{V}^{*} }}{{E_{V} }}V^{*} I_{V}^{*} + \gamma I_{V}^{*} + \gamma \frac{{R_{V}^{*} }}{{R_{V} }}I_{V} + \alpha E_{V}^{*} + \alpha \frac{{I_{V}^{*} }}{{I_{V} }}E_{V} + \gamma \frac{{\left( {I_{V} - I_{V}^{*} } \right)^{2} }}{{I_{V} }} + \alpha \frac{{\left( {E_{V} - E_{V}^{*} } \right)^{2} }}{{E_{V} }}. \\ \end{aligned}$$

It is evident that, $\frac{{{\text{d}}L_{f} }}{{{\text{d}}t}} < 0$ if ${\Pi }_{1} < {\Pi }_{2} .$ However, for $S = S^{*} ,L = L^{*} ,E = E^{*} ,I = I^{*} ,R = R^{*} ,V = V^{*} ,E_{V} = E_{V}^{*} ,I_{V} = I_{V}^{*} ,R_{V} = R_{V}^{*}$ we may write,

12.10 $$\begin{aligned} 0 & = {\Pi }_{1} - {\Pi }_{2} \\ & \Rightarrow \frac{{{\text{d}}L_{f} }}{{{\text{d}}t}} = 0. \\ \end{aligned}$$

We conclude that for the recommended model, the leading compact invariant set in

12.11 $$\left\{ {\left( {S^{*} , L^{*} , E^{*} ,I^{*} ,R^{*} ,V^{*} ,E_{V}^{*} ,I_{V}^{*} ,R_{V}^{*} } \right) \in {\Gamma }:\frac{{{\text{d}}L_{f} }}{{{\text{d}}t}} = 0} \right\}$$

is the endemic equilibrium point $\left\{ {E_{*} } \right\}$. Finally, it is evident that according to Lasalle’s invariance, if ${\Pi }_{1} < {\Pi }_{2}$, $E_{*}$ is globally asymptotically stable in ${\Gamma }$.

### The second derivative of the Lyapunov function (LF)

Generally, the first derivative of LF assists researchers in checking the global stability of the models. However, it helps in knowing crucial information like disease sequence but not well enough to comprehend the variabilities. As a result, second derivative analysis is essential for further information, for example, curvature and sign. The second derivative, we believe, will give more details.

12.12 $$\begin{aligned} \frac{{{\text{d}}\dot{L}_{f} }}{{{\text{d}}t}} & = \frac{{\text{d}}}{{{\text{d}}t}}\left\{ {\left( {1 - \frac{{S^{*} }}{S}} \right)\dot{S} + \left( {1 - \frac{{L^{*} }}{L}} \right)\dot{L} + \left( {1 - \frac{{E^{*} }}{E}} \right)\dot{E} + \left( {1 - \frac{{I^{*} }}{I}} \right)\dot{I} + \left( {1 - \frac{{R^{*} }}{R}} \right)\dot{R} + \left( {1 - \frac{{V^{*} }}{V}} \right)\dot{V}} \right. \\ & \quad + \left( {1 - \frac{{E_{V}^{*} }}{{E_{V} }}} \right)\dot{E}_{V} + \left( {1 - \frac{{I_{V}^{*} }}{{I_{V} }}} \right)\dot{I}_{V} + \left( {1 - \frac{{R_{V}^{*} }}{{R_{V} }}} \right)\dot{R}_{V} \\ & = \left( {\frac{{\dot{S}}}{S}} \right)^{2} S^{*} + \left( {\frac{{\dot{L}}}{L}} \right)^{2} L^{*} + \left( {\frac{{\dot{E}}}{E}} \right)^{2} E^{*} + \left( {\frac{{\dot{I}}}{I}} \right)^{2} I^{*} + \left( {\frac{{\dot{R}}}{R}} \right)^{2} R^{*} + \left( {\frac{{\dot{V}}}{V}} \right)^{2} V^{*} + \left( {\frac{{\dot{E}_{V} }}{{E_{V} }}} \right)^{2} E_{V}^{*} + \left( {\frac{{\dot{I}_{V} }}{{I_{V} }}} \right)^{2} I_{V}^{*} \\ & \quad + \left( {\frac{{\dot{R}_{V} }}{{R_{V} }}} \right)^{2} R_{V}^{*} + \left( {1 - \frac{{S^{*} }}{S}} \right)\ddot{S} + \left( {1 - \frac{{L^{*} }}{L}} \right)\ddot{L} + \left( {1 - \frac{{E^{*} }}{E}} \right)\ddot{E} + \left( {1 - \frac{{I^{*} }}{I}} \right)\ddot{I} + \left( {1 - \frac{{R^{*} }}{R}} \right)\ddot{R} \\ \end{aligned}$$

Here, the second derivative of Eq. (1) is

$$\begin{aligned} \ddot{S} & = - \beta \dot{S}\left( {I + I_{V} } \right) - \beta S\left( {\dot{I} + \dot{I}_{V} } \right) - \delta \dot{S} - l\dot{S} + l_{d} \dot{L} \\ \ddot{L} & = l\dot{S} - \left( {1 - q} \right)\beta \dot{L}\left( {I + I_{V} } \right) - \left( {1 - q} \right)\beta L\left( {\dot{I} + \dot{I}_{V} } \right) - l_{d} \dot{L} \\ \ddot{E} & = \beta \dot{S}\left( {I + I_{V} } \right) + \beta S\left( {\dot{I} + \dot{I}_{V} } \right) + \left( {1 - q} \right)\beta \dot{L}\left( {I + I_{V} } \right) + \left( {1 - q} \right)\beta L\left( {\dot{I} + \dot{I}_{V} } \right) - \alpha \dot{E} \\ \ddot{I} & = \alpha \dot{E} - \gamma \dot{I} \\ \ddot{R} & = \gamma \dot{I} \\ \ddot{V} & = \delta \dot{S} - \left( {1 - \eta } \right)\beta \dot{V}\left( {I + I_{V} } \right) - \left( {1 - \eta } \right)\beta S\left( {\dot{I} + \dot{I}_{V} } \right) \\ \ddot{E}_{V} & = \left( {1 - q} \right)\beta \dot{V}\left( {I + I_{V} } \right) + \left( {1 - q} \right)\beta V\left( {\dot{I} + \dot{I}_{V} } \right) - \alpha \dot{E}_{V} \\ \ddot{I}_{V} & = \alpha \dot{E}_{V} - \gamma \dot{I}_{V} \\ \ddot{R}_{V} & = \gamma \dot{I}_{V} . \\ \end{aligned}$$

Hence,

12.13 $$\begin{aligned} & \frac{{{\text{d}}\dot{L}_{f} }}{{{\text{d}}t}} = \left( {\frac{{\dot{S}}}{S}} \right)^{2} S^{*} + \left( {\frac{{\dot{L}}}{L}} \right)^{2} L^{*} + \left( {\frac{{\dot{E}}}{E}} \right)^{2} E^{*} + \left( {\frac{{\dot{I}}}{I}} \right)^{2} I^{*} + \left( {\frac{{\dot{R}}}{R}} \right)^{2} R^{*} + \left( {\frac{{\dot{V}}}{V}} \right)^{2} V^{*} + \left( {\frac{{\dot{E}_{V} }}{{E_{V} }}} \right)^{2} E_{V}^{*} + \left( {\frac{{\dot{I}_{V} }}{{I_{V} }}} \right)^{2} I_{V}^{*} \\ & \quad + \left( {\frac{{\dot{R}_{V} }}{{R_{V} }}} \right)^{2} R_{V}^{*} + \left( {1 - \frac{{S^{*} }}{S}} \right)\left\{ { - \beta \dot{S}\left( {I + I_{V} } \right) - \beta S\left( {\dot{I} + \dot{I}_{V} } \right) - \delta \dot{S} - l\dot{S} + l_{d} \dot{L}} \right\} \\ & \quad + \left( {1 - \frac{{L^{*} }}{L}} \right)\left\{ {l\dot{S} - \left( {1 - q} \right)\beta \dot{L}\left( {I + I_{V} } \right) - \left( {1 - q} \right)\beta L\left( {\dot{I} + \dot{I}_{V} } \right) - l_{d} \dot{L}} \right\} \\ & \quad + \left( {1 - \frac{{E^{*} }}{E}} \right)\left\{ {\beta \dot{S}\left( {I + I_{V} } \right) + \beta S\left( {\dot{I} + \dot{I}_{V} } \right) + \left( {1 - q} \right)\beta \dot{L}\left( {I + I_{V} } \right) + \left( {1 - q} \right)\beta L\left( {\dot{I} + \dot{I}_{V} } \right) - \alpha \dot{E}} \right\} \\ & \quad + \left( {1 - \frac{{I^{*} }}{I}} \right)\left( {\alpha \dot{E} - \gamma \dot{I}} \right) + \left( {1 - \frac{{R^{*} }}{R}} \right)\gamma \dot{I} \\ & \quad + \left( {1 - \frac{{V^{*} }}{V}} \right)\left\{ {\delta \dot{S} - \left( {1 - \eta } \right)\beta \dot{V}\left( {I + I_{V} } \right) - \left( {1 - \eta } \right)\beta V\left( {\dot{I} + \dot{I}_{V} } \right)} \right\} \\ & \quad + \left( {1 - \frac{{E_{V}^{*} }}{{E_{V} }}} \right)\left\{ {\left( {1 - \eta } \right)\beta \dot{V}\left( {I + I_{V} } \right) + \left( {1 - \eta } \right)\beta V\left( {\dot{I} + \dot{I}_{V} } \right) - \alpha \dot{E}_{V} } \right\} \\ & \quad + \left( {1 - \frac{{I_{V}^{*} }}{{I_{V} }}} \right)\left( {\alpha \dot{E}_{V} - \gamma \dot{I}_{V} } \right) + \left( {1 - \frac{{R_{V}^{*} }}{{R_{V} }}} \right)\gamma \dot{I}_{V} . \\ \end{aligned}$$

and

12.14 $$\begin{aligned} \frac{{{\text{d}}^{2} L_{f} }}{{{\text{d}}t^{2} }} & = {\dot{\Pi }}\left( {S, L,E,I,R, V,E_{V} ,I_{V} ,R_{V} } \right) + \left( {1 - \frac{{S^{*} }}{S}} \right)\left\{ { - \beta \dot{S}\left( {I + I_{V} } \right) - \beta S\left( {\dot{I} + \dot{I}_{V} } \right) - \delta \dot{S} - l\dot{S} + l_{d} \dot{L}} \right\} \\ & \quad + \left( {1 - \frac{{L^{*} }}{L}} \right)\left\{ {l\dot{S} - \left( {1 - q} \right)\beta \dot{L}\left( {I + I_{V} } \right) - \left( {1 - q} \right)\beta L\left( {\dot{I} + \dot{I}_{V} } \right) - l_{d} \dot{L}} \right\} \\ & \quad + \left( {1 - \frac{{E^{*} }}{E}} \right)\left\{ {\beta \dot{S}\left( {I + I_{V} } \right) + \beta S\left( {\dot{I} + \dot{I}_{V} } \right) + \left( {1 - q} \right)\beta \dot{L}\left( {I + I_{V} } \right) + \left( {1 - q} \right)\beta L\left( {\dot{I} + \dot{I}_{V} } \right) - \alpha \dot{E}} \right\} \\ & \quad + \left( {1 - \frac{{I^{*} }}{I}} \right)\left( {\alpha \dot{E} - \gamma \dot{I}} \right) + \left( {1 - \frac{{R^{*} }}{R}} \right)\gamma \dot{I} \\ & \quad + \left( {1 - \frac{{V^{*} }}{V}} \right)\left\{ {\delta \dot{S} - \left( {1 - \eta } \right)\beta \dot{V}\left( {I + I_{V} } \right) - \left( {1 - \eta } \right)\beta V\left( {\dot{I} + \dot{I}_{V} } \right)} \right\} \\ & \quad + \left( {1 - \frac{{E_{V}^{*} }}{{E_{V} }}} \right)\left\{ {\left( {1 - \eta } \right)\beta \dot{V}\left( {I + I_{V} } \right) + \left( {1 - \eta } \right)\beta V\left( {\dot{I} + \dot{I}_{V} } \right) - \alpha \dot{E}_{V} } \right\} \\ & \quad + \left( {1 - \frac{{I_{V}^{*} }}{{I_{V} }}} \right)\left( {\alpha \dot{E}_{V} - \gamma \dot{I}_{V} } \right) + \left( {1 - \frac{{R_{V}^{*} }}{{R_{V} }}} \right)\gamma \dot{I}_{V} . \\ \end{aligned}$$

Finally, replacing the value of $\dot{S}, \dot{L}, \dot{E},\dot{I},\dot{R},\dot{V},\dot{E}_{V} ,\dot{I}_{V} ,\dot{R}_{V}$ in equation $\left( {A1.14} \right)$, we have,

12.15 $$\frac{{{\text{d}}^{2} L_{f} }}{{{\text{d}}t^{2} }} = {\Sigma }_{1} - {\Sigma }_{2} ,$$

where ${\Sigma }_{1}$ and ${\Sigma }_{2}$ are the summation of all positive and negative terms.

Therefore,

$$\frac{{{\text{d}}^{2} L_{f} }}{{{\text{d}}t^{2} }} > 0$$ if $${\Sigma }_{1} > {\Sigma }_{2} ,$$

$$\frac{{{\text{d}}^{2} L_{f} }}{{{\text{d}}t^{2} }} < 0$$ if $${\Sigma }_{1} < {\Sigma }_{2} ,$$

$$\frac{{{\text{d}}^{2} L_{f} }}{{{\text{d}}t^{2} }} = 0$$ if $${\Sigma }_{1} = {\Sigma }_{2} .$$

### Existence and uniqueness

The present sub-section examines the existence and uniqueness of the proposed model’s solution through the concept of classical calculus.

#### Theorem 5:

If $\theta_{i}$ and $\overline{\theta }_{i}$ are the positive constants, then.

(i) $$\forall i \in \left\{ {1, 2, 3, . . ., 9} \right\}$$

12.17 $$\left| {f_{i} \left( {x_{i} , t} \right) - f_{i} \left( {x_{i}{\prime} , t} \right)} \right|^{2} \le \theta_{i} \left| {x_{i} - x_{i}{\prime} } \right|^{2} .$$

(ii) $$\forall \left( {x, t} \right) \in {\mathbb{R}}^{9} \times \left( {0, T} \right)$$

12.18 $$\left| {f_{i} \left( {x_{i} , t} \right)} \right|^{2} \le \overline{\theta }_{i} \left( {1 + \left| {x_{i} } \right|^{2} } \right) or \overline{\theta }_{i} \left| {x_{i} } \right|^{2} .$$

We may represent the current model as follows

$$\begin{aligned} \frac{{{\text{d}}S\left( t \right)}}{{{\text{d}}t}} & = - \beta S\left( t \right)\left( {I\left( t \right) + I_{V} \left( t \right)} \right) - \delta S\left( t \right) - lS\left( t \right) + l_{d} L\left( t \right) = f_{1} \left( {t,S, L,E,I,R, V,E_{V} ,I_{V} ,R_{V} } \right), \\ \frac{{{\text{d}}L\left( t \right)}}{{{\text{d}}t}} & = lS\left( t \right) - \left( {1 - q} \right)\beta L\left( t \right)\left( {I\left( t \right) + I_{V} \left( t \right)} \right) - l_{d} L\left( t \right) = f_{2} \left( {t,S, L,E,I,R, V,E_{V} ,I_{V} ,R_{V} } \right), \\ \frac{{{\text{d}}E\left( t \right)}}{{{\text{d}}t}} & = \beta S\left( t \right)\left( {I\left( t \right) + I_{V} \left( t \right)} \right) + \left( {1 - q} \right)\beta L\left( t \right)\left( {I\left( t \right) + I_{V} \left( t \right)} \right) - \alpha E\left( t \right) = f_{3} \left( {t,S, L,E,I,R, V,E_{V} ,I_{V} ,R_{V} } \right), \\ \frac{{{\text{d}}I\left( t \right)}}{{{\text{d}}t}} & = \alpha E\left( t \right) - \gamma I\left( t \right) = f_{4} \left( {t,S, L,E,I,R, V,E_{V} ,I_{V} ,R_{V} } \right), \\ \frac{{{\text{d}}R\left( t \right)}}{{{\text{d}}t}} & = \gamma I\left( t \right) = f_{5} \left( {t,S, L,E,I,R, V,E_{V} ,I_{V} ,R_{V} } \right), \\ \frac{{{\text{d}}V\left( t \right)}}{{{\text{d}}t}} & = \delta S\left( t \right) - \left( {1 - \eta } \right)\beta V\left( t \right)\left( {I\left( t \right) + I_{V} \left( t \right)} \right) = f_{6} \left( {t,S, L,E,I,R, V,E_{V} ,I_{V} ,R_{V} } \right), \\ \frac{{{\text{d}}E_{V} \left( t \right)}}{{{\text{d}}t}} & = \left( {1 - \eta } \right)\beta V\left( t \right)\left( {I\left( t \right) + I_{V} \left( t \right)} \right) - \alpha E_{V} \left( t \right) = f_{7} \left( {t,S, L,E,I,R, V,E_{V} ,I_{V} ,R_{V} } \right), \\ \frac{{{\text{d}}I_{V} \left( t \right)}}{{{\text{d}}t}} & = \alpha E_{V} \left( t \right) - \gamma I_{V} \left( t \right) = f_{8} \left( {t,S, L,E,I,R, V,E_{V} ,I_{V} ,R_{V} } \right), \\ \frac{{{\text{d}}R_{V} \left( t \right)}}{{{\text{d}}t}} & = \gamma I_{V} \left( t \right) = f_{9} \left( {t,S, L,E,I,R, V,E_{V} ,I_{V} ,R_{V} } \right), \\ \end{aligned}$$

To begin, we will show that the given function $f_{1} \left( {t,S, L,E,I,R, V,E_{V} ,I_{V} ,R_{V} } \right)$ satisfies

12.19 $$|f_{1} \left( {S_{1} , t} \right) - f_{1} \left( {S_{2} , t} \right)|^{2} \le \theta_{1} \left| {S_{1} - S_{2} } \right|^{2} .$$

Therefore,

$$\begin{aligned} |f_{1} \left( {S_{1} , t} \right) - f_{1} \left( {S_{2} , t} \right)|^{2} & = \left| { - \beta \left( {I + I_{V} } \right)\left( {S_{1} - S_{2} } \right) - \delta \left( {S_{1} - S_{2} } \right) - l\left( {S_{1} - S_{2} } \right)} \right|^{2} \\ & = \left| {\left\{ { - \beta \left( {I + I_{V} } \right) - \delta - l} \right\}\left( {S_{1} - S_{2} } \right)} \right|^{2} \\ & \le \left\{ {2\beta^{2} \left( {\left| I \right|^{2} + \left| {I_{V} } \right|^{2} } \right) + 2\delta^{2} + 2l^{2} } \right\}\left| {S_{1} - S_{2} } \right|^{2} \\ & \le \left\{ {2\beta^{2} \left( {\mathop {{\text{sup}}}\limits_{0 \le t \le T} \left| I \right|^{2} + \mathop {{\text{sup}}}\limits_{0 \le t \le T} \left| {I_{V} } \right|^{2} } \right) + 2\delta^{2} + 2l^{2} } \right\}\left| {S_{1} - S_{2} } \right|^{2} \\ & \le \left\{ {2\beta^{2} \left| {\left| {I\left( t \right)} \right|} \right|_{\infty }^{2} + 2\beta^{2} \left| {\left| {I_{V} \left( t \right)} \right|} \right|_{\infty }^{2} + 2\delta^{2} + 2l^{2} } \right\}\left| {S_{1} - S_{2} } \right|^{2} \\ & \le \theta_{1} \left| {S_{1} - S_{2} } \right|^{2} , \\ \end{aligned}$$

where $$\theta_{1} = 2\beta^{2} \left| {\left| {I\left( t \right)} \right|} \right|_{\infty }^{2} + 2\beta^{2} \left| {\left| {I_{V} \left( t \right)} \right|} \right|_{\infty }^{2} + 2\delta^{2} + 2l^{2} .$$

In the same way, the other compartments may be shown to meet the inequality mentioned above.

Secondly, we shall demonstrate that

12.20 $$|f_{1} \left( {S, t} \right)|^{2} \le \overline{\theta }_{1} \left( {1 + \left| S \right|^{2} } \right).$$

Then,

$$\begin{aligned} |f_{1} \left( {S, t} \right)|^{2} & = \left| { - \beta S\left( {I + I_{V} } \right) - \delta S - lS + l_{d} L} \right|^{2} \\ & = \left| {\left\{ { - \beta \left( {I + I_{V} } \right) - \delta - l} \right\}S + l_{d} L} \right|^{2} \\ & \le \left\{ {2\beta^{2} \left( {\left| I \right|^{2} + \left| {I_{V} } \right|^{2} } \right) + 2\delta^{2} + 2l^{2} } \right\}\left| S \right|^{2} + 2l_{d}^{2} \left| L \right|^{2} \\ & \le \left\{ {2\beta^{2} \left( {\mathop {{\text{sup}}}\limits_{0 \le t \le T} \left| I \right|^{2} + \mathop {{\text{sup}}}\limits_{0 \le t \le T} \left| {I_{V} } \right|^{2} } \right) + 2\delta^{2} + 2l^{2} } \right\}\left| S \right|^{2} + 2l_{d}^{2} \mathop {{\text{sup}}}\limits_{0 \le t \le T} \left| L \right|^{2} \\ & \le \left\{ {2\beta^{2} \left( {\left| {\left| {I\left( t \right)} \right|} \right|_{\infty }^{2} + \left| {\left| {I_{V} \left( t \right)} \right|} \right|_{\infty }^{2} } \right) + 2\delta^{2} + 2l^{2} } \right\}\left| S \right|^{2} + 2l_{d}^{2} \left| {\left| {L\left( t \right)} \right|} \right|_{\infty }^{2} \\ & \le \overline{\theta }_{1} \left( {1 + \left| S \right|^{2} } \right), \\ \end{aligned}$$

implies that

$$\frac{{2\beta^{2} \left( {\left| {\left| {I\left( t \right)} \right|} \right|_{\infty }^{2} + \left| {\left| {I_{V} \left( t \right)} \right|} \right|_{\infty }^{2} } \right) + 2\delta^{2} + 2l^{2} }}{{2l_{d}^{2} \left| {\left| {L\left( t \right)} \right|} \right|_{\infty }^{2} }} < 1,$$

where $$\overline{\theta }_{1} = 2l_{d}^{2} \left| {\left| {L\left( t \right)} \right|} \right|_{\infty }^{2} .$$

In the same way, as mentioned earlier, we can also show that inequality holds for the other compartments. To summarize, our system’s solution exists and is unique, as described in [49, 69].

### Positivity and boundedness of solutions (network based model)

#### Lemma 1

Let us assume that the solution.

$({S}_{1},{{L}_{1},{E}_{1},{I}_{1},{R}_{1},V}_{1},{E}_{1}^{V},{I}_{1}^{V},{R}_{1}^{V},\dots ,{S}_{n},{L}_{n},{E}_{n},{I}_{n},{R}_{n},{V}_{n},{E}_{n}^{V},{I}_{n}^{V},{R}_{n}^{V})$ of the proposed system illustrated in Eq. (3.1–3.9) for the given initial condition in $\Pi ,$ where $\Theta \left(0\right)>0.$ Therefore, $0<{S}_{k}\left(t\right)<1, 0<{E}_{k}\left(t\right)<1, 0<{I}_{k}\left(t\right)<1, 0<{R}_{k}\left(t\right)<1, 0<{V}_{k}\left(t\right)<\mathrm{1,0}<{E}_{k}^{V}\left(t\right)<\mathrm{1,0}<{I}_{k}^{V}\left(t\right)<\mathrm{1,0}<{R}_{k}^{V}\left(t\right)<1,and\Theta \left({\text{t}}\right)>0, \forall \mathrm{ t}>0$, where $k=\mathrm{1,2},3,\dots ,n.$

#### Proof

Firstly, we assume that ${\Theta }\left( {\text{t}} \right) > 0,{ }\forall {\text{ t}} > 0$. Then, we can write from Eq. (4)

$$\begin{aligned} {\dot{\Theta }}\left( t \right) & = \frac{1}{\left\langle k \right\rangle }\mathop \sum \limits_{k = 1}^{n} \left( {k - 1} \right)P\left( k \right)\left[ {\dot{I}_{k} \left( t \right) + \dot{I}_{k}^{V} \left( t \right)} \right] \\ & = \frac{1}{\left\langle k \right\rangle }\mathop \sum \limits_{k = 1}^{n} \left( {k - 1} \right)P\left( k \right)\left[ {\alpha E_{k} \left( t \right) - \gamma I_{k} \left( t \right) + \alpha E_{k}^{V} \left( t \right) - \gamma I_{k}^{V} \left( t \right)} \right] \\ & = \frac{1}{\left\langle k \right\rangle }\mathop \sum \limits_{k = 1}^{n} \alpha \left( {k - 1} \right)P\left( k \right)[E_{k} \left( t \right) + E_{k}^{V} \left( t \right)] - \gamma {\Theta }\left( t \right). \\ \end{aligned}$$

Then, we can write,

$$\Theta \left( t \right) = - \gamma \mathop \smallint \limits_{0}^{t} \Theta \left( u \right){\text{d}}u + \mathop \smallint \limits_{0}^{t} \frac{{\alpha \left( {k - 1} \right)P\left( k \right)}}{\left\langle k \right\rangle }\left( {\mathop \sum \limits_{k = 1}^{n} E_{k} \left( u \right) + \mathop \sum \limits_{k = 1}^{n} E_{k}^{V} \left( u \right)} \right){\text{d}}u.$$

As $${\Theta }\left( 0 \right) > 0,$$ and $${\Theta }\left( {\text{t}} \right) > 0,{ }\forall {\text{ t}} > 0.$$

According to the initial condition, ${E}_{k}\left(0\right)\ge 0,{E}_{k}^{V}\left(0\right)\ge 0.$ Then from the continuity of exposed individuals ${E}_{k}\left(t\right), \exists \gamma >0$, which implies that ${E}_{k}\left(0\right)>0$ for $t\in \left(0,\gamma \right).$ Therefore, we have to show that ${E}_{k}\left(t\right)>0 \forall \mathrm{ t}.$ Otherwise, we can locate ${t}_{0}\ge \gamma >0$ such that ${E}_{k}\left({t}_{0}\right)=0$ and ${E}_{k}\left(t\right)>0$ for some $t\in \left(0,{t}_{0}\right).$ Thus, from the third equation, we have,

$$\dot{E}_{k} \left( t \right) \ge 0.$$

It means the fact that ${E}_{k}\left({t}_{0}\right)<0$ for some $t\in \left(0,{t}_{0}\right)$ seems to be in a contradiction. As a result, ${E}_{k}\left(t\right)>0 \forall \mathrm{ t}.$ The following relationship can be derived from equation seven using the positivity of ${V}_{k}\left(t\right), {E}_{k}^{V}\left(t\right),$ and $\Theta \left({\text{t}}\right)$:

$$\dot{E}_{k}^{V} \left( t \right) + \gamma E_{k}^{V} \left( t \right) > 0\;{\text{for}}\;t > 0.$$

Then,

$$E_{k}^{V} \left( t \right) > E_{k}^{V} \left( 0 \right)\exp ( - \gamma t) \ge 0$$

and thus, $$E_{k}^{V} \left( t \right) > 0 \forall t.$$

Analogously, we can write,

$$S_{k} \left( t \right),L_{k} \left( t \right),I_{k} \left( t \right),R_{k} \left( t \right),I_{k}^{V} \left( t \right),R_{k}^{V} \left( t \right) > 0 \forall t.$$

Here, the total population, $N_{k} \left( t \right) = S_{k} \left( t \right) + L_{k} \left( t \right) + E_{k} \left( t \right) + I_{k} \left( t \right) + R_{k} \left( t \right) + V_{k} \left( t \right) + E_{k}^{V} \left( t \right) + I_{k}^{V} \left( t \right) + R_{k}^{k} \left( t \right),$ where $k = 1,2,3, \ldots ,n.$

Then, $$\dot{N}_{k} \left( t \right) = \dot{S}_{k} \left( t \right) + \dot{L}_{k} \left( t \right) + \dot{E}_{k} \left( t \right) + \dot{I}_{k} \left( t \right) + \dot{R}_{k} \left( t \right) + \dot{V}_{k} \left( t \right) + \dot{E}_{k}^{V} \left( t \right) + \dot{I}_{k}^{V} \left( t \right) + \dot{R}_{k}^{k} \left( t \right).$$

Substituting the value of $\dot{S}_{k} \left( t \right),\dot{L}_{k} \left( t \right),\dot{E}_{k} \left( t \right),\dot{I}_{k} \left( t \right),\dot{R}_{k} \left( t \right),\dot{V}_{k} \left( t \right),\dot{E}_{k}^{V} \left( t \right),\dot{I}_{k}^{V} \left( t \right),\dot{R}_{k}^{k} \left( t \right)$ from Eq. (3) in the above equation, we have.

$\dot{N}_{k} \left( t \right) = 0 \forall {\text{ t}} \ge 0,$ which assures that the total population $N_{k} \left( t \right)$ is constant.

Now,

$$N_{k} \left( t \right) = S_{k} \left( t \right) + L_{k} \left( t \right) + E_{k} \left( t \right) + I_{k} \left( t \right) + R_{k} \left( t \right) + V_{k} \left( t \right) + E_{k}^{V} \left( t \right) + I_{k}^{V} \left( t \right) + R_{k}^{k} \left( t \right) = 1 \forall {\text{t}} > 0.$$

As

$$S_{k} \left( t \right) > 0,L_{k} \left( t \right) > 0,E_{k} \left( t \right) > 0,I_{k} \left( t \right) > 0,R_{k} \left( t \right) > 0,V_{k} \left( t \right) > 0,E_{k}^{V} \left( t \right) > 0,I_{k}^{V} \left( t \right) > 0,R_{k}^{k} \left( t \right) > 0;$$

as a result, we accomplish that $0 < S_{k} \left( t \right) < 1, 0 < E_{k} \left( t \right) < 1, 0 < I_{k} \left( t \right) < 1, 0 < R_{k} \left( t \right) < 1, 0 < V_{k} \left( t \right) < 1,0 < E_{k}^{V} \left( t \right) < 1,0 < I_{k}^{V} \left( t \right) < 1,0\left\langle {R_{k}^{V} \left( t \right)\left\langle {1,\;and\;\Theta \left( t \right)} \right\rangle 0, \forall t} \right\rangle 0$,

which concludes the proof.

### Existence and uniqueness of the fractional-order derivative solutions

#### Theorem 6

The current model’s kernels of Eq. (8) accomplish prominent Lipschitz continuity ${LC}_{i}\ge 0, i=1, 2,\dots ,9.$

#### Proof

Suppose,

$$\begin{aligned} {}_{0}^{{ABC}} D_{t}^{\epsilon } S\left( t \right) & = - \beta S\left( t \right)\left( {I\left( t \right) + I_{V} \left( t \right)} \right) - \delta ~S\left( t \right) - lS\left( t \right) + l_{d} L\left( t \right), \\ {}_{0}^{{ABC}} D_{t}^{\epsilon } L\left( t \right) & = lS\left( t \right) - \left( {1 - q} \right)\beta L\left( t \right)\left( {I\left( t \right) + I_{V} \left( t \right)} \right) - l_{d} L\left( t \right), \\ {}_{0}^{{ABC}} D_{t}^{\epsilon } E\left( t \right) & = \beta S\left( t \right)\left( {I\left( t \right) + I_{V} \left( t \right)} \right) + \left( {1 - q} \right)\beta L\left( t \right)\left( {I\left( t \right) + I_{V} \left( t \right)} \right)~ - \alpha E\left( t \right), \\ {}_{0}^{{ABC}} D_{t}^{\epsilon } I\left( t \right) & = \alpha E\left( t \right) - \gamma I\left( t \right), \\ {}_{0}^{{ABC}} D_{t}^{\epsilon } R\left( t \right) & = \gamma I\left( t \right), \\ {}_{0}^{{ABC}} D_{t}^{\epsilon } V\left( t \right) & = \delta ~S\left( t \right) - \left( {1 - \eta } \right)\beta V\left( t \right)\left( {I\left( t \right) + I_{V} \left( t \right)} \right), \\ {}_{0}^{{ABC}} D_{t}^{\epsilon } E_{V} \left( t \right) & = \left( {1 - \eta } \right)\beta V\left( t \right)\left( {I\left( t \right) + I_{V} \left( t \right)} \right) - \alpha E_{V} \left( t \right), \\ {}_{0}^{{ABC}} D_{t}^{\epsilon } I_{V} \left( t \right) & = \alpha E_{V} \left( t \right) - \gamma I_{V} \left( t \right), \\ {}_{0}^{{ABC}} D_{t}^{\epsilon } R_{V} \left( t \right) & = \gamma I_{V} \left( t \right). \\ \end{aligned}$$

Now,

$$\begin{aligned} & |f_{1} \left( {S_{1} , t} \right) - f_{1} \left( {S_{2} , t} \right)\left| = \right| - \beta \left( {I + I_{V} } \right)\left( {S_{1} - S_{2} } \right) - \delta \left( {S_{1} - S_{2} } \right) - l\left( {S_{1} - S_{2} } \right)| \\ & = \left| {\left\{ { - \beta \left( {I + I_{V} } \right) - \delta - l} \right\}} \right|\left( {S_{1} - S_{2} } \right) \\ & \le \left\{ {\left| {\beta \left( {\mathop {{\text{sup}}}\limits_{0 \le t \le T} I + \mathop {{\text{sup}}}\limits_{0 \le t \le T} I_{V} } \right)} \right| + \left| \delta \right| + \left| l \right|} \right\}\left| {\left| {S_{1} - S_{2} } \right|} \right| \\ & \le LC_{1} \left| {\left| {S_{1} - S_{2} } \right|} \right|, \\ \end{aligned}$$

where $LC_{1} = \left\{ {\left| {\beta \left( {\mathop {{\text{sup}}}\limits_{0 \le t \le T} I + \mathop {{\text{sup}}}\limits_{0 \le t \le T} I_{V} } \right)} \right| + \left| \delta \right| + \left| l \right|} \right\},$ which implies that

12.21.1 $$|f_{1} \left( {S_{1} , t} \right) - f_{1} \left( {S_{2} , t} \right)\left| { \le LC_{1} } \right|\left| {S_{1} - S_{2} } \right||.$$

Similarly, we obtain the following inequality if we consider the natural death rates in every compartment.

12.21.2 $$|f_{2} \left( {L_{1} , t} \right) - f_{2} \left( {L_{2} , t} \right)\left| { \le LC_{2} } \right|\left| {L_{1} - L_{2} } \right||,$$

12.21.3 $$|f_{3} \left( {E_{1} , t} \right) - f_{3} \left( {E_{2} , t} \right)\left| { \le LC_{3} } \right|\left| {E_{1} - E_{2} } \right||,$$

12.21.4 $$|f_{4} \left( {I_{1} , t} \right) - f_{4} \left( {I_{2} , t} \right)\left| { \le LC_{4} } \right|\left| {I_{1} - I_{2} } \right||,$$

12.21.5 $$|f_{5} \left( {R_{1} , t} \right) - f_{5} \left( {R_{2} , t} \right)\left| { \le LC_{5} } \right|\left| {R_{1} - R_{2} } \right||,$$

12.21.6 $$|f_{6} \left( {V_{1} , t} \right) - f_{6} \left( {V_{2} , t} \right)\left| { \le LC_{6} } \right|\left| {V_{1} - V_{2} } \right||,$$

12.21.7 $$|f_{7} \left( {E_{{V1}} ,~t} \right) - f_{7} \left( {E_{{V2}} ,~t} \right)\left| { \le LC_{7} } \right|\left| {E_{{V1}} - E_{{V2}} } \right||,$$

12.21.8 $$|f_{8} \left( {I_{{V1}} ,~t} \right) - f_{8} \left( {I_{{V2}} ,~t} \right)\left| { \le LC_{8} } \right|\left| {I_{{V1}} - I_{{V2}} } \right||,$$

12.21.9 $$|f_{9} \left( {R_{{V1}} ,~t} \right) - f_{9} \left( {R_{{V2}} ,~t} \right)\left| { \le LC_{9} } \right|\left| {R_{{V1}} - R_{{V2}} } \right||.$$

### Sensitivity analysis

This part is dedicated to examining the sensitivity analysis pertaining to the basic reproductive number. The dynamics of the proposed model are influenced by several parameters, which are assigned weights according to their sensitivity indices. This study aims to discern the key attributes that exhibit high sensitivity concerning the basic reproduction rate and to illustrate the influence of specific parameters on the trajectory of the basic reproductive number, either increasing or decreasing. Therefore,

$$\frac{{\partial R_{0} }}{\partial q} = - \frac{\beta }{\gamma } < 0.$$

The impact of the lockdown maintenance factor on the basic reproduction number is seen to be negative. Hence, it can be inferred that the degree of adherence to lockdown measures or the enhancement of lockdown effectiveness leads to a decrease in the overall disease burden.

$$\frac{{\partial R_{0} }}{\partial \eta } = - \frac{\beta }{\gamma } < 0.$$

From the above equation, it is crystal clear that a vaccine’s efficacy has a negative effect on the basic reproduction number. Therefore, a highly effective vaccine is crucial to eradicate any disease from any society.

### The partial rank correlation coefficients (PRCCs)

To determine the parameters of the model that have the most effect on the chosen response function, insightful uncertainty, and sensitivity analysis have been applied using the Latin Hypercube Sampling technique and partial rank correlation coefficients (PRCCs). The sensitivity analysis process involved defining a range (lower and upper bounds) and distribution for each model parameter. Subsequently, the matrix of parameter values was systematically split, and ${R}_{0}$ of the model, along with corresponding PRCCs, was computed to gauge each parameter's contribution to uncertainty and variability in ${R}_{0}$. Parameters exhibiting high PRCC values near $-1$ or $+1$ were deemed highly correlated with the response function. Negative (positive) PRCC values indicated negative (positive) associations with the response function. A positive sensitivity index for a model parameter implied that an increase in the parameter would raise the basic reproduction number and vice versa. Table 2 outlines critical parameters in our proposed model, including transmission $(\beta )$, vaccine efficacy $(\eta )$, recovery $(\gamma )$, and lock-down maintenance $(q)$ rates. Examining Fig. 12, it becomes evident that tracking down infected individuals and strictly adhering to safety measures can decrease the disease's transmission rate $(\beta )$. The value of $\eta =-0.451558$ signifies that ${R}_{0}$ decreases by 45% when $\eta$ increases by 100%. Similar explanations apply to the remaining two parameters.

**Table 2:** The sensitivity index is associated with each model parameter

Parameter	Sensitivity index
$\beta$	0.638029
$\eta$	− 0.451558
$\gamma$	− 0.881569
$q$	− 0.440801
